# *Wwox* Deficiency Causes Downregulation of Prosurvival ERK Signaling and Abnormal Homeostatic Responses in Mouse Skin

**DOI:** 10.3389/fcell.2020.558432

**Published:** 2020-10-27

**Authors:** Ying-Tsen Chou, Feng-Jie Lai, Nan-Shan Chang, Li-Jin Hsu

**Affiliations:** ^1^Institute of Basic Medical Sciences, College of Medicine, National Cheng Kung University, Tainan, Taiwan; ^2^Department of Dermatology, Chimei Medical Center, Tainan, Taiwan; ^3^Center for General Education, Southern Taiwan University of Science and Technology, Tainan, Taiwan; ^4^Institute of Molecular Medicine, College of Medicine, National Cheng Kung University, Tainan, Taiwan; ^5^Graduate Institute of Biomedical Sciences, College of Medicine, China Medical University, Taichung, Taiwan; ^6^Department of Medical Laboratory Science and Biotechnology, College of Medicine, National Cheng Kung University, Tainan, Taiwan

**Keywords:** WWOX, tumor suppressor, keratinocyte proliferation, keratinocyte differentiation, stem cells, adipocytes

## Abstract

Deficiency of tumor suppressor WW domain-containing oxidoreductase (WWOX) in humans and animals leads to growth retardation and premature death during postnatal developmental stages. Skin integrity is essential for organism survival due to its protection against dehydration and hypothermia. Our previous report demonstrated that human epidermal suprabasal cells express WWOX protein, and the expression is gradually increased toward the superficial differentiated cells prior to cornification. Here, we investigated whether abnormal skin development and homeostasis occur under *Wwox* deficiency that may correlate with early death. We determined that keratinocyte proliferation and differentiation were decreased, while apoptosis was increased in *Wwox*^–/–^ mouse epidermis and primary keratinocyte cultures and *WWOX*-knockdown human HaCaT cells. Without WWOX, progenitor cells in hair follicle junctional zone underwent massive proliferation in early postnatal developmental stages and the stem/progenitor cell pools were depleted at postnatal day 21. These events lead to significantly decreased epidermal thickness, dehydration state, and delayed hair development in *Wwox*^–/–^ mouse skin, which is associated with downregulation of prosurvival MEK/ERK signaling in *Wwox*^–/–^ keratinocytes. Moreover, *Wwox* depletion results in substantial downregulation of dermal collagen contents in mice. Notably, *Wwox*^–/–^ mice exhibit severe loss of subcutaneous adipose tissue and significant hypothermia. Collectively, our knockout mouse model supports the validity of WWOX in assisting epidermal and adipose homeostasis, and the involvement of prosurvival ERK pathway in the homeostatic responses regulated by WWOX.

## Introduction

WW domain-containing oxidoreductase (WWOX) has been initially known as a proapoptotic tumor suppressor (Chang et al., 2007; Lo et al., 2015; Schrock and Huebner, 2015). WWOX loss promotes tumor growth and cancer progression through allowing metastatic cancer cells to survive in WWOX-positive normal microenvironment (Chou et al., 2019a, b). Expression of WWOX protein is essential for cell apoptosis induced by anti-cancer agents, UV irradiation, genotoxic stress, or ectopic p53 (Chang et al., 2003, 2005a; Abu-Remaileh et al., 2015; Chen et al., 2015; Janczar et al., 2017; Huang and Chang, 2018). Recently, *WWOX* gene has also been considered as a risk factor for human Alzheimer’s disease (Kunkle et al., 2019). Ser14 phosphorylation and accumulation of WWOX protein in the brain contribute to the Alzheimer’s memory loss in mice (Chang et al., 2015; Lee et al., 2017). WWOX also controls many biological functions, including bubbling cell death and genomic stability (Abu-Remaileh et al., 2015; Chen et al., 2015), suggesting that WWOX plays an important role in normal cellular/physiological homeostasis maintenance. Moreover, the first WW domain of WWOX participates in protein-protein interactions in multiple signaling pathways (Abu-Odeh et al., 2014; Liu et al., 2018; Saigo et al., 2018). Crosstalk of WWOX with many signal pathways also implies the involvement of WWOX in development and stemness maintenance (Chen et al., 2019).

Human newborns carrying homozygous mutations or deletions of *WWOX* gene exhibit growth retardation, severe neurological disorders, and early death (Abdel-Salam et al., 2014; Mallaret et al., 2014; Valduga et al., 2015). *Wwox* knockout (*Wwox*^–/–^) mice display similar symptoms and die within 4 weeks after birth (Aqeilan et al., 2007; Ludes-Meyers et al., 2009). Previous studies have demonstrated that lipid metabolism, steroidogenesis, bone development and metabolism, and central nervous system are defective in *Wwox*^–/–^ mice (Aqeilan et al., 2008; Aqeilan et al., 2009; Hussain et al., 2019; Cheng et al., 2020). The deficits in whole-body *Wwox* knockout mice may recapitulate the crucial clinical features of human pathological conditions (Cheng et al., 2020). However, the main cause leading to early death of WWOX-deficient newborns is still unclear. WWOX protein is abundant in many types of human epithelial tissues (Nunez et al., 2006). Mice carrying conditional depletion of *Wwox* in basal epithelial cells under the control of *keratin-5* (K5) promoter also die prematurely for unknown reasons (Ferguson et al., 2012), suggesting a vital role of WWOX in regulating proper epithelial functions and survival.

In normal human skin, WWOX protein is expressed in the innermost proliferative basal layer of epidermal keratinocytes and hair follicles (HFs), and its protein expression pattern displays an upregulation in a gradient toward outer differentiated suprabasal layers (Lai et al., 2005; Nunez et al., 2006). The maintenance of epidermal homeostasis depends on the coordination of basal cell proliferative capacity and basal-to-suprabasal differentiation processes. This phenomenon ensures epidermal barrier functions to protect the body from UV irradiation, pathogen infection and uncontrolled water loss for survival. The HFs, developed through the downward growth of epidermal basal keratinocytes into the dermis, contribute to thermoregulation. The maturation and regeneration of HFs largely rely on the self-renewal capacity of HF stem cells (HFSCs) and progenitors. Along with the epidermal stem cells, HFSCs are also crucial for epidermal self-renewal and homeostasis maintenance (Blanpain and Fuchs, 2009; Yi and Fuchs, 2010). Our previous study showed that WWOX is essential for UV-induced apoptosis in human HaCaT keratinocytes and squamous cell carcinoma cells (Lai et al., 2005). However, whether WWOX controls epidermal homeostasis and hair growth remains largely unclear. Moreover, the subcutaneous fat is involved in hair growth regulation and serves as a thermal insulator to prevent heat loss and maintain body temperature (Alexander et al., 2015). Body temperature reduction is a marker for imminent death in aging or microbial infection of mice (Ray et al., 2010; Gavin and Satchell, 2017). It has been shown that *Wwox*^–/–^ mice exhibit lower serum lipid levels (Aqeilan et al., 2009). Notably, the influence of WWOX deficiency on the homeostasis of subcutaneous fat and body temperature remains to be investigated.

In this study, we showed that *Wwox* depletion in mice caused decreased proliferation and differentiation but increased apoptosis in keratinocytes. In addition, epidermal and HF stem cell numbers were reduced in *Wwox*^–/–^ mice. The dysregulations were partly due to decreased prosurvival extracellular signal-regulated kinase (ERK) signaling in *Wwox*^–/–^ keratinocytes. Strikingly, *Wwox*^–/–^ mice lost more than 95% of the subcutaneous fat layer in dermal tissues. Collectively, *Wwox*^–/–^ mice display aberrant epidermal functions, delayed hair morphogenesis and hypothermia, which may partly contribute to the life-threatening conditions in pre-weaning mice.

## Materials and Methods

### Animals and Cell Culture

Whole-body *Wwox* gene knockout mice were generated as described previously (Tsai et al., 2013; Cheng et al., 2020). This study was carried out in accordance with the approved protocols for animal use from the Institutional Animal Care and Use Committee of National Cheng Kung University. Human HaCaT cell line was maintained in Dulbecco’s Modified Eagle’s Medium (GE-Hyclone, Logan, UT, United States) supplemented with 10% fetal bovine serum (FBS). HaCaT cells were cultured in keratinocyte serum-free medium or EpiLife medium containing 30 or 60 μM calcium ion, respectively, with the supplementation of human keratinocyte growth supplements (Life Technologies, Carlsbad, CA, United States) for reversion to a basal cell phenotype before differentiation induction. For differentiation and stratification induction, 1 × 10^6^ cells were seeded in a 10-cm dish for 48 h and 2 mM calcium chloride was added to the cells. Primary keratinocytes were isolated and cultured according to a previous protocol (Jensen et al., 2010). Briefly, mouse skin was collected, and the connective tissue and subcutaneous fat were scraped-off. Skin tissues were then placed in 8 ml of dispase solution (BD biosciences) at 4°C for 6 h and/or 0.25% trypsin (Gibco) at 37°C for 2.5∼3 h with epithelium facing up. The disassociated skin epithelium was separated from the dermis, and the cells were further digested with 0.25% trypsin. After wash, the cells were then passed through a 40-μm cell strainer to remove debris or clumps. The isolated primary keratinocytes were cultured in keratinocyte growth medium (Cell Applications, Inc., San Diego, CA, United States) supplemented with 10% FBS overnight. The medium was replaced with serum-free keratinocyte growth medium fully supplemented with keratinocyte growth factors and antibiotics (Cat. No. 131-500; Cell Applications, Inc.) on the next day. All cells were maintained at 37°C in 5% CO_2_.

### Histological, Collagen, and Nile Red Staining

Mouse skin tissues were fixed in 10% formalin/PBS and embedded in paraffin. Four-μm sections were deparaffinized in xylene, rehydrated in serial concentrations of ethanol (100, 95, 80, and 70%), and then stained with hematoxylin and eosin (H&E) solutions. For detecting collagen fibers, tissue sections were stained with hematoxylin, followed by a Sirius red solution (0.1% Sirius red in saturated aqueous solution of picric acid) for 1 h. For detecting intracellular lipid droplets, tissue sections were embedded using an OCT compound (Sakura, Tokyo, Japan). Nile red solution was prepared as described previously (Chou et al., 2005). Ten-μm frozen skin tissue sections were stained in Nile red solution for 10 min in dark, and then treated with 4% potassium hydroxide subsequently. Lipid droplets in skin tissues were examined under a fluorescence microscope (Olympus, Tokyo, Japan).

### Immunohistochemical and Immunofluorescent Staining and Terminal Deoxynucleotidyl Transferase dUTP Nick End Labeling (TUNEL) Assay

Paraffin-embedded skin tissue sections were deparaffinized in xylene and rehydrated in serial concentrations of ethanol. Antigen retrieval was performed using microwave for 25 min in 10 mM citric buffer (pH = 6.0). For CD34 staining, skin tissues were embedded in an OCT compound (Sakura, Tokyo, Japan). The frozen tissue sections were air-dry, washed with distilled water and fixed in 10% formalin/PBS for 10 min at room temperature. For immunohistochemical staining, endogenous peroxidase was quenched using 3% H_2_O_2_. After blocking in a solution containing 1% bovine serum albumin and 0.1% saponin for 30 min at room temperature, the tissue sections were incubated in the primary antibody solution at 4°C overnight. For loricrin, fatty acid binding protein 4 (FABP4), CD34 and keratin 15 (K15) staining, the sections were incubated with horseradish peroxidase (HRP)-conjugated secondary antibody (Cell Signaling, Danvers, MA, United States) for 1 h at room temperature. For Ki67 staining, a polymeric HRP-conjugated secondary antibody (Novolink Polymer, Leica Biosystems, Newcastle upon Tyne, United Kingdom) and AEC substrate solution (DAKO, Carpinteria, CA, United States) were used. For ERK1/2, Thr202/Tyr204-phosphorylated ERK (pERK) and K15 staining, a polymer detection system (REAL EnVision, DAKO, Carpinteria, CA, United States) was used according to the manufacturer’s instructions. The staining results were examined under a light microscope (Olympus BX51, Tokyo, Japan).

For immunofluorescence staining, tissue sections were incubated with primary antibody solution at 4°C overnight, and then with an Alexa 488- or Alexa 594-conjugated secondary antibody (Invitrogen) for 1 h at room temperature. TUNEL assay was performed according to the manufacturer’s instructions (Millipore, Temecula, CA, United States). For staining the nuclei, tissue sections were incubated with a 4′,6-diamidino-2-phenylindole (DAPI) staining solution at room temperature for 5 min. The tissue samples were visualized under a fluorescence microscope (Olympus BX61, Tokyo, Japan) or a laser-scanning confocal microscope (Olympus FV1000, Tokyo, Japan).

### Toluidine Blue Staining and Transmission Electron Microscopy

Mouse skin tissues were fixed in 4% glutaraldehyde/0.1M cacodylate buffer (pH 7.4) at 4°C overnight. After post-fixation using 1% OsO_4_, samples were dehydrated and embedded in resin (Embed 812, EMS). Semithin sections (0.5 μm) were stained with 1% toluidine blue/2% sodium borate for 2 min, washed with distilled water and examine under a light microscope (Olympus BX51, Tokyo, Japan). Ultrathin sections (90 nm) were prepared, stained with 2% uranyl acetate for 20 min and 4% lead citrate for 3 min, and examined under a transmission electron microscope (JEOL JEM-1200EX, Tokyo, Japan) as described previously (Cheng et al., 2020).

### Western Blot

Cellular protein extracts were prepared using a lysis buffer containing 1% Nonidet P-40, 0.5% Tween 20, 0.1% SDS, 10 mM Na_4_P_2_O_7_, 10 mM Na_3_VO_4_, 10 mM NaF, and 1:20 dilution of protease inhibitor cocktail (Sigma, St. Louis, MO, United States) in PBS. To generate tissue protein extracts, a tissue homogenizer TissueLyser was used (Thermo Fisher Scientific). Proteins were quantified using a Bradford protein assay kit (Bio-Rad). Equal amounts of proteins were heated at 95°C in a sample buffer (250 mM Tris-HCl pH = 6.8, 500 mM dithiothreitol, 10% SDS, 0.1% bromophenol blue, 50% glycerol) for 10 min, and SDS-PAGE performed for protein separation (Tsai et al., 2013). The proteins were transferred to a piece of polyvinylidene difluoride membrane. After blocking with a skim milk-containing buffer, the membrane was hybridized with an indicated primary antibody in the blocking solution at 4°C overnight. After wash, HRP-conjugated secondary antibody was used (Cell signaling, Danvers, MA, United States) for probing. An enhanced chemiluminescence assay kit (Millipore) was used for protein detection.

### Flow Cytometric Analysis

For propidium iodide (PI) staining, cells were fixed in a 70% ethanol/PBS solution, followed by staining in a PI staining solution (40 μg/ml PI and 100 μg/ml RNase in PBS) at room temperature for 30 min. For 5-bromo-2′-deoxyuridine (BrdU) staining, primary keratinocytes were fixed in a cytofix/cytoperm solution (BD) overnight and stained using a BrdU staining kit according to the manufacture’s instructions (BD). The fluorescence intensity was determined using a FACScalibur (BD), and data analyzed using BD CellQuest Pro software.

### Antibodies

The antibodies used in this study were as follows: K5 (1:1000, Covance #PRB-160P), keratin 10 (K10) for immunohistochemistry (1:100, Covance #MS-159), K10 for western blot (1:10000, Abcam #ab76318), K15 (1:100, Thermo Fisher Scientific #MS-1068; 1:50, Santa Cruz #sc-47697), loricrin (1:1000, Covance #PRB-145P), Ki67 (1:150, DAKO #M7249), p63 (1:50, Santa Cruz #sc-8431), E-cadherin (1:40, Cell Signaling #3195), ERK1/2 (1:50, Santa Cruz #sc-514302), p-ERK (Thr202/Tyr204, 1:200, Cell Signaling #4370), CD34 (1:50, eBioscience #14-0341-82) and FABP4 (1:200, Abcam #ab13979). For detecting WWOX expression, an anti-WWOX antibody recognizing both human and mouse proteins was used (Chang et al., 2001, 2005b).

### Skin Hydration State and Body Temperature Measurement

Skin hydration state was measured on shaved mouse dorsal skin using the MPA 5 system equipped with a Corneometer 825 probe (Courage + Khazaka electronic GmbH, Cologne, Germany). For measuring transepidermal water loss, mouse dorsal skin was shaved using hair removal cream, and the MPA 5 system equipped with a Tewameter TM300 probe was used (Courage + Khazaka electronic GmbH). Body temperature was determined on the shaved mouse dorsal skin surface using fiber-optic temperature sensors (Neoptix/Reflex signal conditioner, Quebec City, QC, Canada).

### Statistical Analysis and Quantification Analysis

Statistical analysis was performed using Prism 6 software (GraphPad Software, San Diego, CA, United States). The measurements of epidermal thickness, dermal thickness, hair length, and hair width were performed using Image-Pro Plus 6 (Media Cybernetics, Rockville, MD, United States). Mean fluorescence and red color intensities on mouse skin surface were analyzed using ImageJ software (Wayne Rasband, National Institutes of Health). Quantitative analysis of immunohistochemical staining was performed using ImageJ plugin IHC Profiler (Varghese et al., 2014).

## Results

### *Wwox* Loss Results in Reduced Epidermal Integrity

**FIGURE 1:**
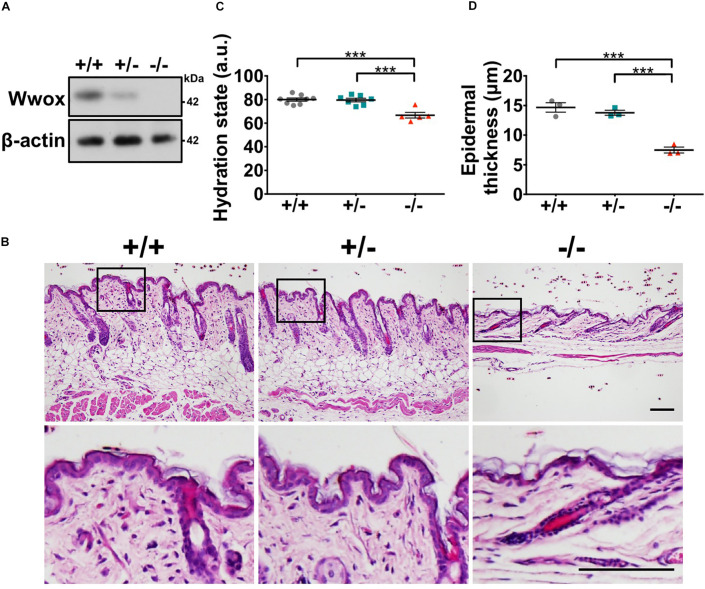
*Wwox* knockout leads to reduced epidermal integrity. **(A)** Western blot analysis for WWOX protein expression in *Wwox*^+/+^, *Wwox*^+/–^, and *Wwox*^–/–^ mouse skin at P21. β-Actin was used as a loading control. **(B)** H&E staining results of *Wwox*^+/+^, *Wwox*^+/–^, and *Wwox*^–/–^ mouse skin tissue sections at P21. Scale bar = 100 μm. The lower panels are enlarged images from the boxed areas in the upper panels. **(C)** Mouse skin hydration status at ∼3-week-old (*Wwox*^+/+^, *n* = 8; *Wwox*^+/–^, *n* = 7; *Wwox*^–/–^, *n* = 5). **(D)** Measurement of mouse epidermal thickness (*n* = 3). Data are presented as mean ± standard error of the mean (SEM). No animals were excluded from the analysis and no randomization was used in this study. One-way ANOVA and *post hoc* Tukey tests were performed for statistical analysis. ***P < 0.001.

To investigate whether WWOX deficiency affects skin development, homeostasis and functions, whole-body knockout mouse models for *Wwox* gene were used in this study (Cheng et al., 2020). The absence of WWOX protein expression in *Wwox*^–/–^ mouse skin was verified (Figure 1A). *Wwox*^–/–^ mouse skin exhibited morphological abnormalities (Figure 1B). However, tumors or severe clinical cutaneous disorders, such as blisters, were not developed (Figure 1B). When assessing the skin barrier function, we did not find increased transepidermal water loss in *Wwox*^–/–^ mice as the lipid layer was still maintained on the *Wwox*^–/–^ epidermal surface (Supplementary Figure S1). However, the hydration levels of *Wwox*^–/–^ epidermis were significantly reduced (Figure 1C). The *Wwox*^–/–^ epidermal thickness was also significantly decreased at postnatal day (P) 21 (Figures 1B,D). These results suggest that WWOX is required for maintenance of normal epidermal function and homeostasis.

### *Wwox* Is Associated With Keratinocyte Differentiation and Stratification

**FIGURE 2:**
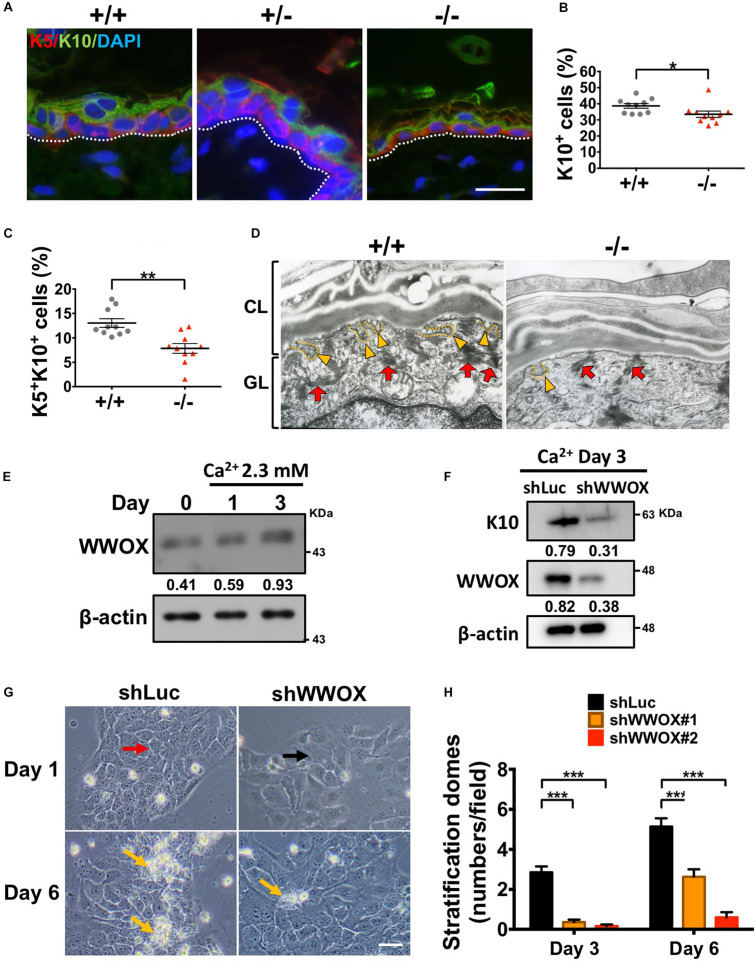
WWOX loss leads to defective keratinocyte differentiation and stratification. **(A)** Immunofluorescence staining of *Wwox*^+/+^, *Wwox*^+/–^, and *Wwox*^–/–^ mouse tissue sections for the expression of K5 (red) and K10 (green). DAPI was used for the staining of nuclei (blue). Dotted lines indicate the basement membrane. Scale bar = 20 μm. The percentages of epidermal K10^+^ **(B)** and K5^+^/K10^+^ cells **(C)** were quantified (*n* = 10). Student’s *t*-test was performed for statistical analysis. **(D)** Keratohyalin granules (red arrows) and fusion of granules with the cell membrane (yellow arrowheads) were examined by transmission electron microscopy. CL, cornified layer; GL, granular layer. Magnification: 30,000x. **(E)** WWOX protein expression in HaCaT cells was determined by western blot analysis after calcium chloride treatment for 1 and 3 days. β-Actin was used as a loading control. The numbers indicate the ratio of WWOX to β-actin protein levels in cells. **(F)** Western blot anslysis of WWOX and K10 protein expression in calcium chloride-treated control (shLuc) or WWOX-knockdown (shWWOX) HaCaT cells. β-actin was used as a loading control. **(G)** shLuc or shWWOX HaCaT cells were treated with 2 mM calcium chloride for 1 and 6 days. The cells with clear cell-cell junctions (red arrow) were observed in the control, but not shWWOX HaCaT cells (black arrow) at day 1. More stratification domes (yellow arrows) were observed in the control cells after treatment for 6 days. Scale bar = 50 μm. **(H)** HaCaT cells were treated with 2 mM calcium chloride for 3 or 6 days to induce differentiation. The stratification domes in the shLuc and shWWOX HaCaT cells were quantified. At least 15 fields were quantified from two and three independent experiments at day 3 and 6, respectively. Data are presented as mean ± SEM. One-way ANOVA and *post hoc* Tukey tests were performed for statistical analysis. *P < 0.05, **P < 0.01, ***P < 0.001.

The multilayered structure of epidermis is established and maintained by the stepwise keratinocyte differentiation and stratification processes (Blanpain and Fuchs, 2009). Proper epidermal terminal differentiation is also required for skin hydration maintenance. Therefore, the consequences of WWOX deficiency on epidermal differentiation were investigated. By immunofluorescent staining, less K10^+^ cells and K5^+^K10^+^ early differentiating progenitors were examined in *Wwox*^–/–^ epidermis (Figures 2A–C). The numbers of loricrin-positive cells that represent late-differentiated keratinocytes were decreased in a majority of *Wwox*^–/–^ mice (Supplementary Figure S2A). However, there were no statistical differences between wild-type and knockout mice due to *Wwox*^–/–^ epidermal atrophy (Supplementary Figure S2B). As shown in Supplementary Figure S2A, loricrin was distributed loosely in *Wwox*^–/–^ epidermal tissues. Moreover, the amount of keratohyalin granules and their fusion with the cell membrane were reduced in *Wwox*^–/–^ epidermal keratinocytes, indicating defective keratinocyte terminal differentiation in *Wwox*^–/–^ epidermis (Figure 2D).

To confirm whether WWOX is required for keratinocyte differentiation and stratification, *in vitro* culture of human HaCaT keratinocytes in serum-free keratinocyte medium was also tested (Deyrieux and Wilson, 2007). The addition of Ca^2+^ to subconfluent HaCaT cell culture has been shown to induce cell-cell association as well as keratinocyte differentiation and stratification (Hennings et al., 1980; Poumay and Pittelkow, 1995; Deyrieux and Wilson, 2007). WWOX protein expression was increased by Ca^2+^ treatment in HaCaT cells in a time-dependent manner (Figure 2E). Lentivirus-mediated knockdown of WWOX protein expression in HaCaT cells suppressed Ca^2+^-induced expression of keratinocyte differentiation marker K10 (Figure 2F). Upon Ca^2+^-induced differentiation and stratification, control cells became cuboidal and formed cell-cell junctions at day 1 (red arrow; Figure 2G). In contrast, the cell boundaries between adjacent WWOX-knockdown cells were less clear after *in vitro* culture for 1 day (black arrow; Figure 2G). Moreover, the control cells formed many stratification domes (piled-up cell clusters) after treatment of Ca^2+^ for 6 days, while much fewer stratification domes were observed in WWOX-knockdown cells (yellow arrows; Figures 2G,H). Together, these results suggest that WWOX is required for keratinocyte differentiation and stratification.

### WWOX Loss Leads to Reduced Proliferation and Survival in Keratinocytes

**FIGURE 3:**
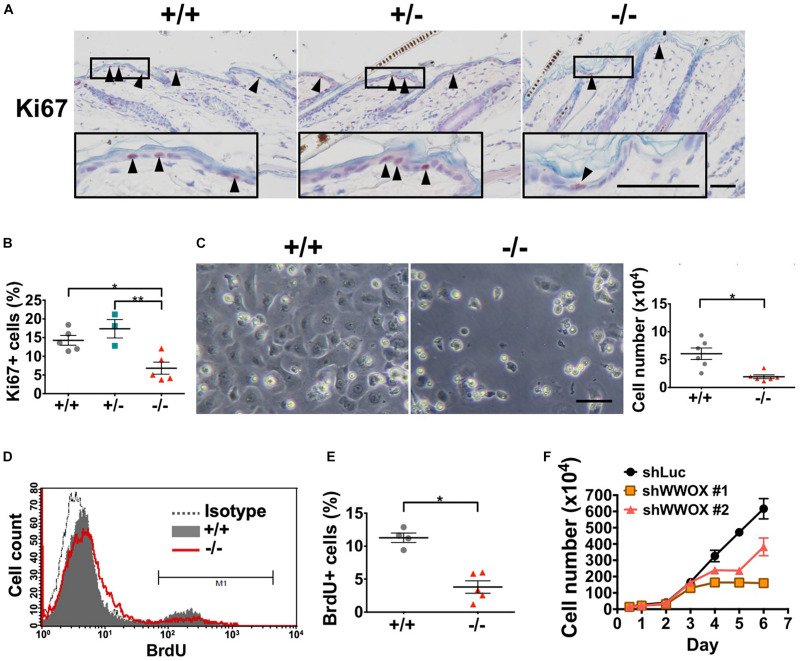
WWOX loss suppresses keratinocyte proliferation at P20. **(A)** Immunohistochemical staining of *Wwox*^+/+^, *Wwox*^+/–^, and *Wwox*^–/–^ mouse epidermal tissue sections for Ki67 (arrowheads). Nuclei were stained with hematoxylin. The lower panels are enlarged images from the boxed areas. Scale bar = 50 μm. **(B)** Quantification of epidermal Ki67-positive cells is shown (*Wwox*^+/+^, *n* = 5; *Wwox*^+/–^, *n* = 3; *Wwox*^–/–^, *n* = 5). One-way ANOVA and *post hoc* Tukey tests were performed for statistical analysis. **(C)** Representative phase-contrast microphotographs of cultured primary keratinocytes isolated from *Wwox*^+/+^ and *Wwox*^–/–^ mice at ∼P20. The right panel indicates the cell numbers quantified using a hemacytometer after *in vitro* culture for 3 days. Scale bar = 10 μm. Paired *t*-test was performed for statistical analysis. **(D)** BrdU incorporation of the cultured primary *Wwox*^+/+^ and *Wwox*^–/–^ mouse keratinocytes was analyzed by flow cytometry. **(E)** Quantification of BrdU incorporation data is shown (*Wwox*^+/+^, *n* = 4; *Wwox*^–/–^, *n* = 5). Paired *t*-test was performed for statistical analysis. **(F)** Growth curves of control sh*Luc* and sh*WWOX* HaCaT cells. All data are presented as mean ± SEM. *P < 0.05, **P < 0.01.

The establishment and maintenance of epidermal multi-differentiated structure are highly dependent on the basal cell proliferative capacities (Blanpain and Fuchs, 2009). As basal keratinocytes express WWOX protein (Nunez et al., 2006), keratinocyte proliferation and cell growth under *Wwox* deficiency were examined. We determined that *Wwox*^–/–^ mouse epidermis had significantly decreased Ki67-positive keratinocytes at P20 (Figures 3A,B). In primary *Wwox*^–/–^ mouse epidermal keratinocyte cultures, the cell numbers were significantly reduced as compared with the control group after *in vitro* culture for 3 days (Figure 3C). A decreased incorporation of BrdU into *Wwox*^–/–^ primary keratinocytes was also observed, indicating the reduction of proliferative activities in these cells (Figures 3D,E). PI staining for cell cycle analysis revealed that the percentages of *Wwox*^–/–^ primary keratinocytes in S and G2/M phases at day 3 were also reduced (Supplementary Figure S3). The role of WWOX in regulating keratinocyte growth was also confirmed using HaCaT cells. In comparison with control cells, WWOX knockdown resulted in significant suppression of HaCaT cell growth (Figure 3F).

We assessed whether the decreased epidermal thickness in *Wwox*^–/–^ mouse skin occurs as a result of increased cell death. Cell cycle analysis using flow cytometry revealed a higher percentage of *Wwox*^–/–^ mouse primary keratinocytes in sub G0 phase than the control, suggesting that *Wwox*^–/–^ keratinocytes tend to undergo apoptosis (Supplementary Figure S3). TUNEL assay further confirmed the higher percentage of apoptotic cells in *Wwox*^–/–^ mouse epidermis than that of the wild-type control at P21 (1.77% in knock-out and 0.77% in wild-type) (Supplementary Figures S4A,B), suggesting that epidermal cell apoptosis is facilitated by WWOX loss.

### *Wwox* Ablation Delays Postnatal HF Morphogenesis

**FIGURE 4:**
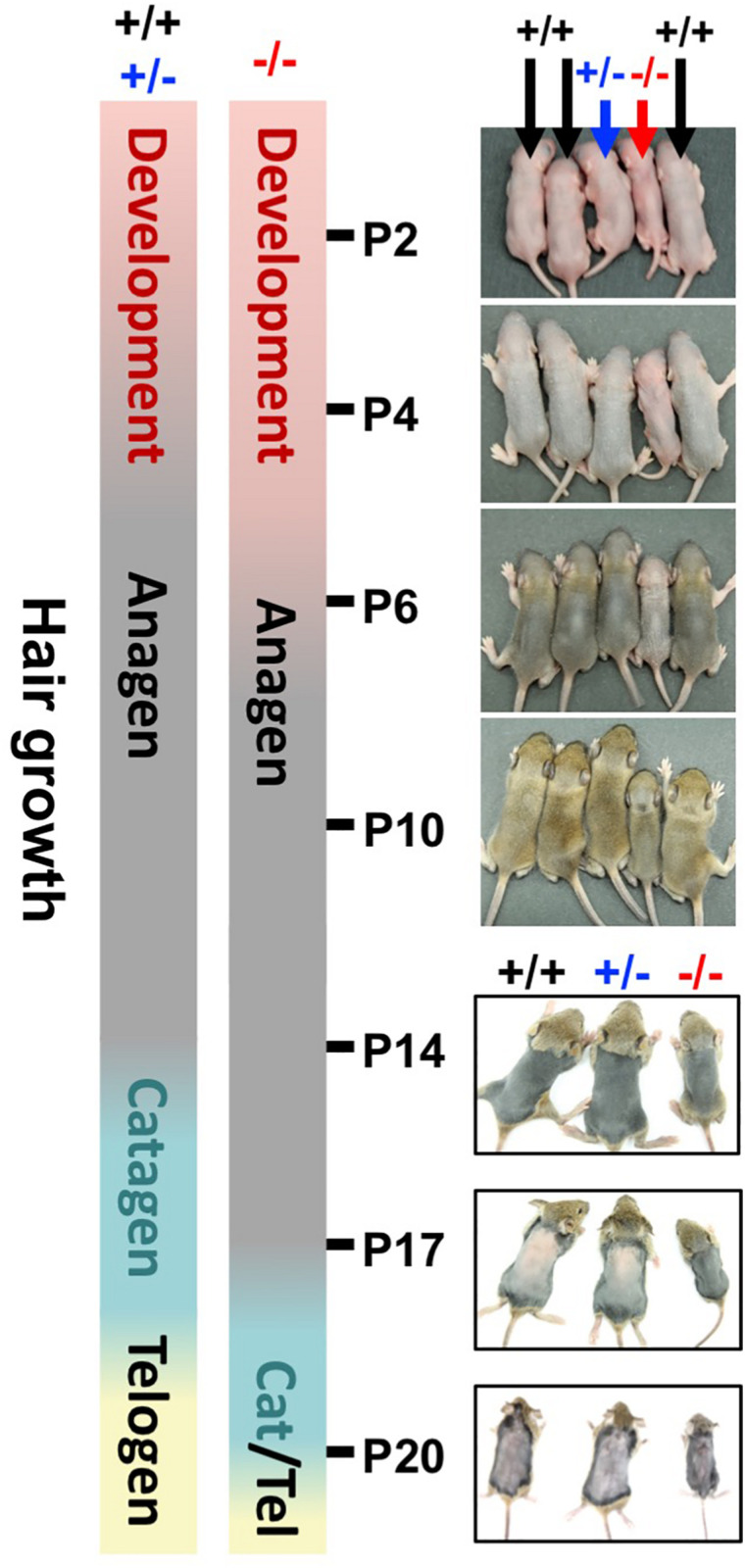
*Wwox* depletion delays hair growth in mice. The bars indicate the timetables of the first hair cycle progression in *Wwox*^+/+^, *Wwox*^+/–^, and *Wwox*^–/–^ mice. The developing skin of mouse littermates was photographed continually from P2 to P20 (black arrows: wild type; blue arrows: heterozygous knockout; red arrows: homozygous knockout).

**FIGURE 5:**
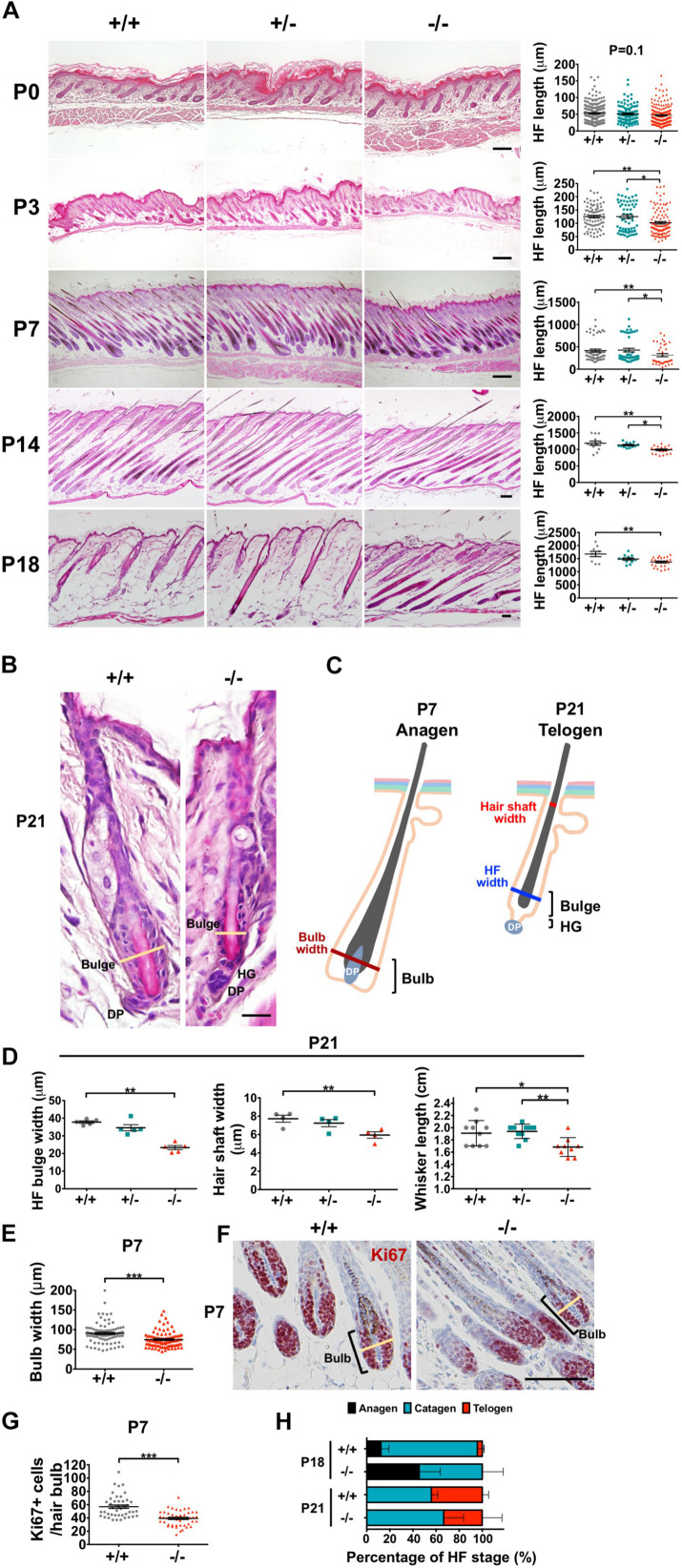
*Wwox* deficiency leads to hair follicle atrophy in mice after birth. **(A)** Representative dorsal skin tissue sections of *Wwox*^+/+^, *Wwox*^+/–^, and *Wwox*^–/–^ mice at birth (P0), P3, P7, P14, and P18 are shown (H&E stain). The quantification results of mouse anagen HF length are shown in the right panels. The numbers of HFs and mice analyzed (HFs/mice) were as the follows: at P0, *Wwox*^+/+^ (224/5), *Wwox*^+/–^ (108/3), and *Wwox*^–/–^ (185/4); at P3, *Wwox*^+/+^ (125/3), *Wwox*^+/–^ (61/3), and *Wwox*^–/–^ (107/3); at P7, *Wwox*^+/+^ (52/4), *Wwox*^+/–^ (42/4), and *Wwox*^–/–^ (36/3); at P14, *Wwox*^+/+^ (12/3), *Wwox*^+/–^ (12/3), and *Wwox*^–/–^ (20/4); at P18, *Wwox*^+/+^ (9/4), *Wwox*^+/–^ (12/4), and *Wwox*^–/–^ (24/4). Scale bars: P0 = 50 μm; P3 = 100 μm; P7 = 200 μm; P14 = 100 μm; P18 = 50 μm. **(B)** Representative telogen HF images of dorsal skin tissue sections from *Wwox*^+/+^ and *Wwox*^–/–^ mice at P21 (H&E stain). The yellow lines indicate HF bulge width. HG, hair germ; DP, dermal papilla. Scale bar = 20 μm. **(C)** An illustration of anagen and telogen HFs. **(D)** The widths of telogen HF bulge (left, *n* = 5) and hair shaft (middle, *n* = 4) were analyzed using serial skin tissue sections from mice at P21. The hair fiber length of mouse whisker (right, *Wwox*^+/+^, *n* = 10; *Wwox*^+/–^, *n* = 10; *Wwox*^–/–^, *n* = 9) was measured at P21. **(E)** HF bulb widths were analyzed using serial skin tissue sections from *Wwox*^+/+^ and *Wwox*^–/–^ mice at P7 (92 HFs/3 mice). Student’s *t*-test was performed for statistical analysis. **(F)** Immunohistochemical staining of *Wwox*^+/+^ and *Wwox*^–/–^ mouse HF bulb sections for Ki67. Nuclei were stained with hematoxylin. The yellow lines indicate HF bulb width. Scale bar = 100 μm. **(G)** Quantification of hair bulb Ki67-positive cells at P7 is shown. Student’s *t*-test was performed for statistical analysis. **(H)** The percentages of anagen, catagen and telogen HFs were examined in *Wwox*^+/+^ and *Wwox*^–/–^ mouse skin sections at P18 (*n* = 4) and P21 (*n* = 4). One-way ANOVA and *post hoc* Tukey tests were performed for statistical analysis. Data analyzed using properly sectioned tissue samples and whiskers are presented as mean ± SEM. One-way ANOVA and *post hoc* Tukey tests were performed for statistical analysis. *P < 0.05, **P < 0.01, ***P < 0.001.

HFs are formed by the downward growth of epidermal basal cells. HFSCs reside in HF bulges and are able to replenish the epidermal keratinocytes and maintain homeostasis (Blanpain and Fuchs, 2009). Since both epidermal basal cells and HF keratinocytes express WWOX protein (Nunez et al., 2006), the effect of WWOX loss on HF growth was investigated by examining a litter of mouse pups continuously after birth. *Wwox*^+/+^ and *Wwox*^+/–^ mice exhibited furs approximately at P4. However, *Wwox*^–/–^ mice grew a first coat of hair after P6 (Figure 4). Compared with the *Wwox*^+/+^ and *Wwox*^+/–^ littermates, the relative fur length of *Wwox*^–/–^ mice was shorter at P10 (Supplementary Figure S5). Histological analysis revealed shorter HF length in *Wwox*^–/–^ mice from P3 to P21 (Figures 5A,B). Moreover, hair fibers were thinner and shorter in *Wwox*^–/–^ mice at P21, as determined by the measurement of HF bulge and hair shaft widths and whisker length (Figures 5B–D). To study whether the deficiency of hair shaft production in *Wwox*^–/–^ HFs is due to defective HF matrix, we detected an average smaller bulb width and a significantly reduced number of Ki67-positive proliferating cells in the hair bulbs of *Wwox*^–/–^ mice at P7 (Figures 5E–G). However, the HF number and length showed no differences among *Wwox*^+/+^, *Wwox*^+/–^, and *Wwox*^–/–^ mice at birth or the embryonic stages (Figure 5A and Supplementary Figure S6).

Starting from P4, mouse HFs undergo cyclic growth and degeneration with a phased series known as anagen, catagen, and telogen (Alonso and Fuchs, 2006). To assess the phase progression of the first hair cycle, mouse skin color and appearance were examined (Muller-Rover et al., 2001). After shaving, the skin color of *Wwox*^+/+^ and *Wwox*^+/–^ mice was brownish-black at P14 and turned pink at P17, indicating that their skin HFs were growing in anagen phase at P14 and entering into catagen phase at P17 (Figure 4 and Supplementary Figure S5). In contrast, *Wwox*^–/–^ HFs were still growing at P17 and entering into catagen around P20 (Figure 4 and Supplementary Figure S5). To study the progression of first hair cycle in mice, histological analysis was performed. As most HFs entered catagen phase in wild-type mouse back skin at P18, few HFs reached the first telogen phase in these mice (Figure 5H). However, a large number of *Wwox*^–/–^ HFs are still in anagen phase at P18 (Figure 5H). Compared with the wild-type control mice, *Wwox*^–/–^ mice had more catagen HFs but less telogen HFs at P21, although no statistically significant differences were found (Figure 5H). Whether *Wwox*^–/–^ mouse hair follicles have shorter catagen phase is unclear. In catagen phase, cells in the lower region of HFs undergo apoptosis (Alonso and Fuchs, 2006). At P18, TUNEL-positive cells were found in the catagen HFs of *Wwox*^+/+^ and *Wwox*^+/–^ mice but not in *Wwox*^–/–^ anagen HFs (Supplementary Figure S4C). The TUNEL results revealed that wild-type HFs reached the first telogen at P18 and *Wwox*^–/–^ HFs at P21 (Supplementary Figure S4C). Our data suggest that HF morphogenesis as well as the phase progression of the first hair growth cycle are delayed by *Wwox* ablation.

### *Wwox* Sustains the Maintenance of Stem Cell Pool in Mouse Skin

**FIGURE 6:**
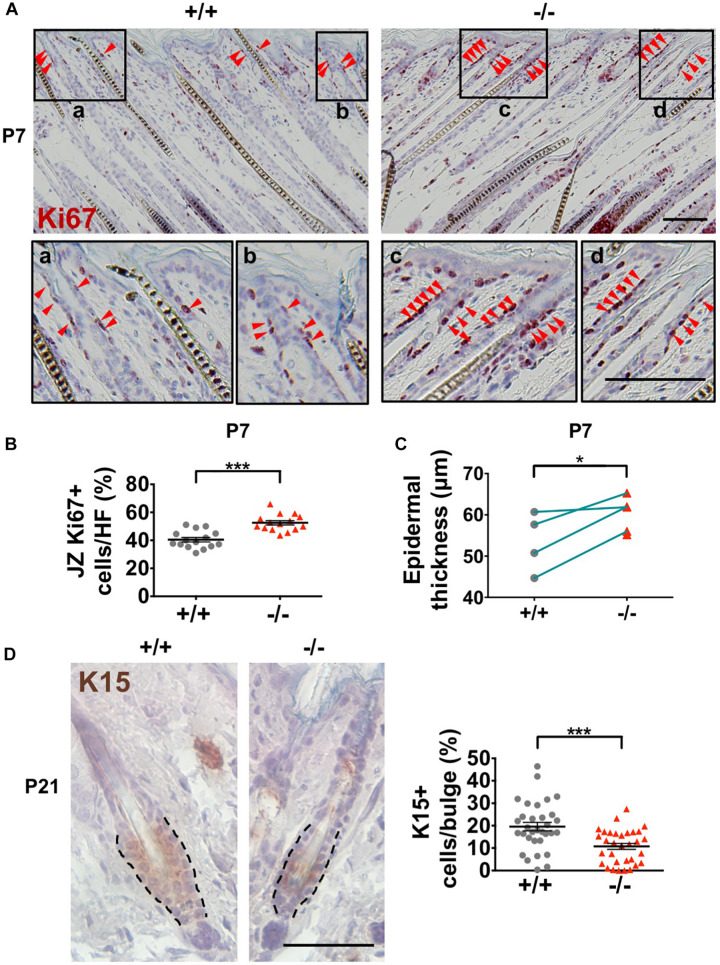
Decreased HF stem cell numbers in *Wwox*^–/–^ skin. **(A)** Immunohistochemical staining of *Wwox*^+/+^ and *Wwox*^–/–^ mouse skin tissue sections for Ki67 at P7. Red arrowheads indicate Ki67^+^ cells. The lower panels are enlarged images from the boxed areas. Scale bar = 100 μm. **(B)** Quantification of Ki67-positive cells in the JZ of *Wwox*^+/+^ and *Wwox*^–/–^ mouse HFs at P7 is shown. **(C)** Measurement of mouse epidermal thickness at P7 (*n* = 4). The green lines indicate paired *Wwox*^+/+^ and *Wwox*^–/–^ mice from the same litter. **(D)** Immunohistochemical staining of *Wwox*^+/+^ and *Wwox*^–/–^ mouse skin tissue sections for K15 at P21. Dotted lines depict the HF bulge regions. The quantification results of K15^+^ cells in the HF bulge region are shown in the right panel. The numbers of HFs and mice analyzed (HFs/mice) were *Wwox*^+/+^ (32/3) and *Wwox*^–/–^ (39/3). Scale bar = 50 μm. Data are presented as mean ± SEM. Student’s *t*-test was performed for statistical analysis. *P < 0.05. ***P < 0.001.

The HFSCs give rise to all epidermal cell lineages (Senoo, 2013; Hsu et al., 2014). The completely specified HFSCs appear at birth in mouse skin. After undergoing initial expansion to accommodate HF development and hair growth, HFSCs become quiescent in early postnatal developmental stages (Nowak et al., 2008). Loss of HFSC proliferative activities disrupts postnatal HF development (Wang et al., 2013). In contrast, over-activation of HFSC proliferation results in depletion of stem cell pools as well as dysregulation of epidermal homeostasis (Niessen et al., 2013; Ali et al., 2016). *Wwox*^–/–^ mouse epidermis showed no increases in cell apoptosis during the delayed postnatal HF morphogenesis (Supplementary Figure S4C). However, increased cell proliferation (Ki67-positive) was detected in the HF junctional zone (JZ) in *Wwox*^–/–^ mice between P3 and P8 as compared to that of the wild-type mice (Figures 6A,B). The JZ progenitors have been shown to commit to epidermal lineages (Kretzschmar and Watt, 2014). The enhanced cell proliferation in JZ also caused transient thickening of the epidermal tissues in *Wwox*^–/–^ mice at P7 (Figure 6C). Next, we analyzed whether the increased cell proliferation in JZ at the early postnatal stage causes a change in HFSC populations. Staining of the bulge HFSC markers K15 and CD34 revealed a reduction in the number of bulge HFSCs in *Wwox*^–/–^ mouse skin tissues at P21 as compared with the controls (Figure 6D and Supplementary Figure S7), suggesting that *Wwox* is involved in the maintenance of HFSC pool. Whether the enhanced proliferative activity of JZ progenitors in *Wwox*-deficient HFs at early postnatal days causes gradual loss of stem cell functionality and leads to HFSC exhaustion at a later stage remains further investigation.

### *Wwox* Loss Reduces E-Cadherin, ERK, and p63 Expression in Keratinocytes

E-cadherin belongs to the family of cell adhesion molecules and is important for the formation of adherens junctions between the adjacent cells. The association of cytosolic β-catenin with E-cadherin at adherens junctions stabilizes E-cadherin expression in epithelial cells and the structure of intercellular junctions (Nelson and Nusse, 2004; Voronkov and Krauss, 2013), and play crucial roles in epidermal and HF development, stem cell renewal, and tissue homeostasis. Depletion of E-cadherin expression in the epidermis and HFs results in alteration of epidermal proliferation and differentiation, defects in HF development, and loss of stem cells. Herein, our data revealed that E-cadherin protein expression was decreased in the epidermis of *Wwox*^–/–^ mice (Supplementary Figure S8).

**FIGURE 7:**
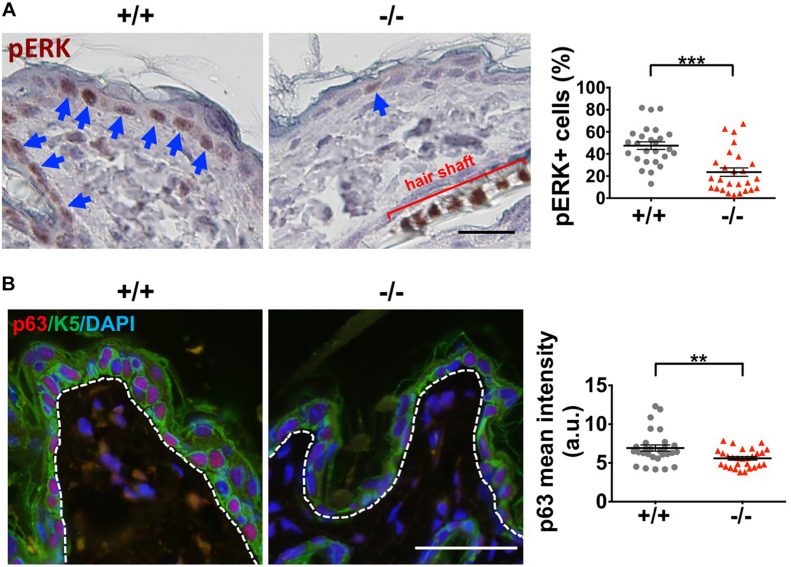
*Wwox* knockout leads to decreased ERK and p63 expression in keratinocytes. **(A)** Immunohistochemical staining of *Wwox*^+/+^ and *Wwox*^–/–^ mouse epidermal tissue sections for pERK was examined under a light microscopy. The blue arrows indicate pERK-positive keratinocytes. Quantification results of pERK expression are shown in the right panel (25 regions/3 mice). All data are presented as mean ± SEM. Student’s *t*-test was performed for statistical analysis. Scale bar = 20 μm. ***P < 0.001. **(B)** Immunofluorescence staining of *Wwox*^+/+^ and *Wwox*^–/–^ mouse skin tissue sections for p63 (red) and K5 (green) at P21 was examined under a confocal microscopy. DAPI was used for the staining of nuclei (blue). Dotted lines depict the location of basement membrane. The quantification results are shown in the right panel (28 regions/4 mice). All data are presented as mean ± SEM. Scale bar = 20 μm. **P < 0.01.

Moreover, *Wwox*^–/–^ epidermal keratinocytes expressed significantly reduced levels of pERK and total ERK1/2 protein (Figure 7A and Supplementary Figure S9, respectively). ERK protein phosphorylation in MAPK signaling is essential for cell proliferation. Therefore, the regulation of keratinocyte proliferation by WWOX may be partly through MAPK signaling. In agreement with a previous study (Salah et al., 2013), we determined reduced expression of epidermal progenitor marker p63 in *Wwox* knockout mouse epidermis (Figure 7B), suggesting that progenitor cell depletion occurs in the skin of *Wwox* knockout mice. Together, our results suggest that *Wwox* absence leads to compromised epidermal homeostasis, stem cell maintenance, and hair development that may be partly due to the loss of E-cadherin, ERK and p63 expression.

### *Wwox* Knockout Mice Have Reduced Dermal Collagen Content, Lack Subcutaneous Fat, and Display Hypothermia

**FIGURE 8:**
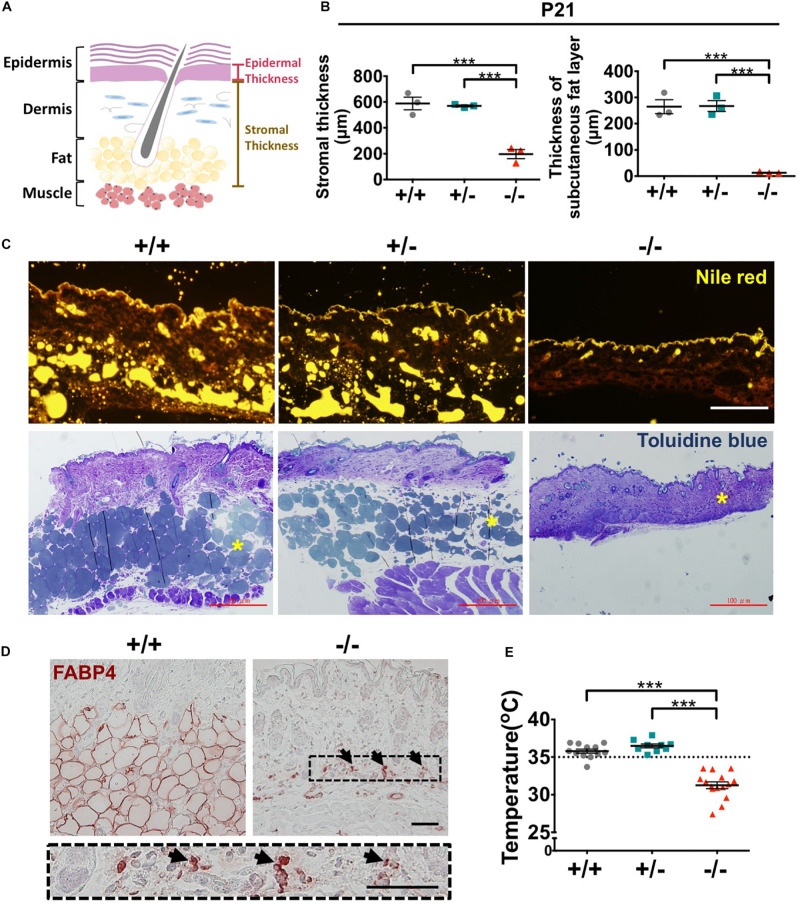
*Wwox*^–/–^ mice lack subcutaneous fat and display hypothermia. **(A)** A schematic diagram of epidermal and stromal thickness measurement. **(B)** Quantification of mouse stromal and subcutaneous fat layer thickness at P21 (*n* = 3). **(C)** Subcutaneous fat was detected by Nile red staining of frozen mouse skin tissue sections (upper panel) and toluidine blue staining of EMbed 812-embedded skin samples (lower panel). The yellow stars in the lower panel indicate subcutaneous fat layer. Scale bar = 100 μm. **(D)** Immunohistochemical staining of *Wwox*^+/+^ and *Wwox*^–/–^ mouse dermal tissue sections for FABP4. The lower panel is an enlarged image from the boxed area in *Wwox*^–/–^ tissue section and the arrows indicate adipocytes. Scale bar = 50 μm. **(E)** Measurement of mouse skin surface temperature (*Wwox*^+/+^, *n* = 12; *Wwox*^+/–^, *n* = 9; *Wwox*^–/–^, *n* = 15). All data are presented as mea ± SEM. Statistical analysis was performed using one-way ANOVA and *post hoc* Tukey tests. ***P < 0.001.

The stromal tissue beneath the epidermis is composed of dermis and subcutaneous fat layers (Figure 8A). Signaling from the stromal cells is crucial for HF morphogenesis and epidermal homeostasis (Hsu et al., 2014; Fujiwara et al., 2018; Rognoni and Watt, 2018). Therefore, changes in stromal composition in mouse skin by *Wwox* gene ablation were examined. Histological analysis revealed that the stromal thickness of *Wwox*^–/–^ mice was dramatically decreased (left panel in Figure 8B). The thickness of dermis was significantly reduced in *Wwox*^–/–^ mice (Supplementary Figure S10A). By transmission electron microscopy, we found that the collagen content was reduced in the dermal layer of *Wwox*^–/–^ mice (Supplementary Figure S10B). Sirius red staining results also revealed that the collagen fibers in *Wwox*^–/–^ mouse dermis were thinner than those of the control littermates (Supplementary Figure S10C).

Decreased serum lipid level has been detected in *Wwox*^–/–^ mice (Aqeilan et al., 2007). We found that *Wwox*^–/–^ mouse subcutaneous fat layer lost at P21 by Nile red and toluidine blue staining (Figure 8C). Immunohistochemical staining for FABP4, a mature adipocyte marker, showed that only a few FABP4-positive cells have remained in *Wwox*^–/–^ mouse dermal tissues (Figure 8D). These cells shrank and did not contain lipid droplets (black arrows; Figure 8D). Subcutaneous fat is vital for thermal regulation and maintaining body temperature (Alexander et al., 2015). Indeed, the average body temperature of *Wwox*^–/–^ mice was significantly decreased (31.3°C in knockout and 35.8°C in wild-type mice; Figure 8E). Together, ablation of *Wwox* gene in mice leads to the change of stromal environment in the dermis that may compromise epidermal and HF development and tissue homeostasis. The loss of subcutaneous fat causes hypothermia in *Wwox*^–/–^ mice.

## Discussion

In summary, our knockout mouse model provides the validity of WWOX in supporting epidermal homeostasis, HF development and stem cell maintenance. Lack of WWOX leads to reduced keratinocyte proliferation and differentiation, as reflected by significant reduction in epidermal thickness, dehydration state, and delayed hair development. Downregulation of E-cadherin expression and prosurvival MEK/ERK signaing pathway is implicated for the aberrant development under the WWOX-deficient setting *in vivo*. Human patients or mice carrying double allele mutation or deletion of *WWOX/Wwox* gene display growth retardation and early death (Aqeilan et al., 2008; Ferguson et al., 2012; Mallaret et al., 2014; Tabarki et al., 2015). Dramatic loss of adipose tissue in the subcutaneous area accounts for hypothermia and severe illness in newborns.

In addition, evidence was provided in this study that WWOX is involved in epidermal homeostasis and HF development through regulating keratinocyte proliferation, differentiation and stratification, and stem cell maintenance. It is likely as a result of orchestrating MEK/ERK signaling pathway. WWOX is an adaptor protein in MEK/ERK signaling pathway (Lin et al., 2011). MEK/ERK signaling has been reported to be regulated by WWOX (Lin et al., 2011; Chen et al., 2019). Activation of MEK/ERK signaling is critical for keratinocyte growth and differentiation and stem cell functions. Conceivably, WWOX is required to control keratinocyte proliferation and differentiation.

Nunez et al. (2006) along with the data shown in Human Protein Atlas^1^ have indicated that epidermal basal and suprabasal cells have weak to moderate WWOX expression. Lai et al. (2005) showed that the intensity of WWOX protein expression is increased toward superficial layers of human epidermis. Using an *in vitro* keratinocyte differentiation model, we determined that WWOX protein expression was increased after differentiation induction by treatment of Ca^2+^ in HaCaT cells. In addition, WWOX knockdown suppressed Ca^2+^-induced differentiation marker K10 expression and stratification dome formation. Together, these findings suggest that WWOX plays an important role in the regulation of epidermal keratinocyte differentiation and stratification. In the case that subconfluent HaCaT cells are cultured with a low concentration of Ca^2+^, the cells undergo proliferation but not differentiation (Bikle et al., 2012). Treatment with a high concentration of Ca^2+^ induces cell cycle exit, differentiation, and formation of desmosomes, tight junctions and adherens junctions in HaCaT cells (Bikle et al., 2012). In the absence of WWOX, reduced E-cadherin expression at the cell junctions and delayed cell-cell contact formation were observed. How WWOX regulates E-cadherin protein expression in epidermal keratinocytes is unknown. Whether the increased WWOX protein expression during keratinocyte differentiation is required for the assembly of adherens junctions for keratinocyte stratification remains to be investigated. Moreover, p63 is an essential molecule for initiating basal to spinous transition of epidermal keratinocytes (Blanpain and Fuchs, 2009; Wu et al., 2012). WWOX has been found to interact with ΔNp63α (Salah et al., 2013). Downregulation of p63 protein level was detected in *Wwox*^–/–^ mouse epidermal keratinocytes in this study. However, it is unclear whether WWOX controls keratinocyte differentiation through p63.

Substantial evidences have suggested that the control of cell growth and apoptosis by WWOX may depend on cell types, such as their tissue origin, differentiation state or differences between normal, benign and malignant cells (Chang et al., 2007; Nowakowska et al., 2014). In coincidence with its tumor suppressor function, overexpression of WWOX protein in malignant lung, breast, pancreatic, cervical, and prostate cancer cells suppresses cell growth and increases cell apoptosis (Fabbri et al., 2005; Iliopoulos et al., 2007; Nakayama et al., 2008; Qu et al., 2013; Lin et al., 2015). In contrast, in low-grade invasive HT29 colon adenocarcinoma cells, increased WWOX expression promotes cell proliferation but inhibits apoptosis (Nowakowska et al., 2014). WWOX has been suggested to have pro-survival functions (Aqeilan et al., 2008; Abdel-Salam et al., 2014; Mallaret et al., 2014). Ludes-Meyers et al. (2009) showed that *Wwox* knockout mice exhibit severe metabolic acidosis and leukopenia and succumb to early death by 3 weeks of age. Targeted ablation of *Wwox* in mice leads to impaired steroidogenesis and reduced GABA-ergic inhibitory interneuron numbers (Aqeilan et al., 2009; Hussain et al., 2019). *Wwox* knockout causes a reduction in mouse bone osteoprogenitor cell growth but does not affect mammary gland cell proliferation (Aqeilan et al., 2008; Ferguson et al., 2012). Using a keratin 5-Cre-mediated knockout mouse model, Ferguson et al. (2012) showed that ablation of *Wwox* in mammary epithelium leads to impaired mammary branching morphogenesis. These findings suggest that WWOX is a protein with pleiotropic functions. A certain amount of WWOX expression may be necessary for maintaining normal physiological functions in cells. However, it remains largely unclear whether the phosphorylation status of WWOX protein in cells affects its functions. The distinct function of WWOX in various cell types may also be manipulated by the expression or mutation of WWOX-binding proteins, such as p53, BRCA, or Ras. How WWOX controls cell fates in coordination with these factors and their regulated signaling remains to be determined.

Both epidermis and HFs are squamous epithelium composed of multi-layered keratinocytes (Blanpain and Fuchs, 2009; Yi and Fuchs, 2010). The basal keratinocytes in the innermost layer are highly proliferative and undifferentiated cells. When these cells are committed to differentiation, they exit cell cycle and begin to differentiate (Fuchs and Raghavan, 2002). Downregulation of cell proliferation in basal keratinocytes has been shown to induce cell differentiation (Asare et al., 2017). A significant reduction in proliferative activity was determined in *Wwox*^–/–^ mouse keratinocytes at ∼P20. However, we did not detect an increase of K5^+^K10^+^ double-positive differentiated progenitor cells in *Wwox*^–/–^ mouse epidermis (Figure 2A). On the contrary, decreased keratinocyte differentiation was examined in *Wwox*^–/–^ mouse epidermis and WWOX-knockdown HaCaT cells. One possibility to achieve it is that WWOX binds to particular proteins involved in both proliferation and differentiation in keratinocytes. However, the molecular mechanisms by which WWOX regulates both keratinocyte proliferation and differentiation remain to be investigated.

The integrity of HFSC niche is maintained by the balance between quiescence and activation status of cells. To produce rapidly cycling and highly proliferative transient amplifying cells, the quiescent stem cells in the epidermal basal layer may undergo asymmetric division (Hsu et al., 2014; Kretzschmar and Watt, 2014). Previous studies have shown that depletion of E-cadherin expression in cells disturbs spindle orientation and cellular polarity during cell division (Wang et al., 2018). In the present study, downregulation of E-cadherin expression was determined in *Wwox*^–/–^ mouse epidermal keratinocytes. However, whether loss of WWOX expression in self-renewing epithelium causes aberrant cellular polarity in epidermal basal cells and disturbs asymmetric division is unknown. The regulation of cell fate, differentiation, and HFSC dynamics for maintaining tissue homeostasis by WWOX remains to be investigated.

Using a conditional knockout mouse model for *Wwox* gene in K5-positive basal cells, Ferguson et al. (2012) found that the mouse epidermis exhibits a normal two-layer structure. However, these conditional *Wwox* knockout mice die within 4 months after birth (Ferguson et al., 2012). Herein, an *in vitro* growth failure of isolated primary *Wwox* knockout mouse epidermal keratinocytes was examined, suggesting an intrinsic defect due to *Wwox* loss in these cells. Although the macro-morphology of epidermis remains intact in these conditional knockout mice (Ferguson et al., 2012), loss of *Wwox* expression in the epidermal keratinocytes might affect cell proliferation, differentiation and adhesion, and impair skin barrier functions. Interestingly, the epidermal thickness was transiently increased at P7 (Figure 6C) but decreased at P21 in the whole-body *Wwox* knockout mice (Figure 1D). A temporary increase in proliferating progenitors observed in the JZ at P7 may lead to a gradual loss of quiescent HFSCs at the later stages. Whether stem cell exhaustion and aberrant keratinocyte proliferation and differentiation cause subsequent abnormality in skin barrier function and death in the keratinocyte-specific *Wwox* knockout mice is unclear. Both collagen and adipose tissues are important for epidermal tissue homeostasis and HF growth (Rognoni and Watt, 2018; Kruglikov et al., 2019). The significant reduction in dermal collagen and subcutaneous fat contents in whole-body *Wwox* knockout mice may result in decreased generation of signals for supporting keratinocyte growth and differentiation and cause more severe phenotypes in the skin.

*Wwox* knockout mice have been reported to exhibit epileptic seizure symptoms and hypoglycemia, which are highly related to mortality (Ludes-Meyers et al., 2009; Abdel-Salam et al., 2014; Cheng et al., 2020). In addition, hypothermia caused by the loss of subcutaneous fat may also lead to early death of *Wwox* knockout mice because low body temperature has been considered as a valid marker for imminent death (Ray et al., 2010; Mai et al., 2018). Downregulation of peroxisome-proliferator activated receptor-γ was examined in *Wwox*^–/–^ mouse subcutaneous fat layer at P7 (data not shown). Whether WWOX regulates adipocyte maturation and function is unclear. Previous studies have revealed that *Wwox* ablation alters glucose and lipid metabolism and causes defective steroidogenesis in mice (Aqeilan et al., 2009; Abu-Remaileh and Aqeilan, 2014; Abu-Remaileh et al., 2019). Nevertheless, whether the fat loss in whole-body *Wwox* knockout mice is a consequence of metabolic dysregulation remains to be investigated.

## Conclusion

Genetic ablation of *Wwox* causes severe dermal and epidermal disorders as well as hypothermia in mice. Most importantly, *Wwox* knockout mouse models may recapitulate the key pathological features of human diseases due to loss or dysfunction of WWOX. Using whole-body or conditional knockout mouse models, future studies will be needed to investigate the molecular mechanisms by which WWOX controls body temperature and regulates HFSC maintenance and keratinocyte proliferation and differentiation for maintaining tissue homeostasis in the skin.

## Data Availability Statement

The original contributions presented in the study are included in the article/Supplementary Material, further inquiries can be directed to the corresponding author.

## Ethics Statement

The animal study was reviewed and approved by Institutional Animal Care and Use Committee of National Cheng Kung University.

## Author Contributions

Y-TC performed the experiments, analyzed the results, and prepared the manuscript. F-JL performed transmission electron microscopy and contributed to the discussion. N-SC contributed to the discussion and reviewed the manuscript. L-JH supervised the work and edited the manuscript. All authors contributed to the article and approved the submitted version.

## Conflict of Interest

The authors declare that the research was conducted in the absence of any commercial or financial relationships that could be construed as a potential conflict of interest.

## Footnotes

1 www.proteinatlas.org
