# Supplementary figures and images for: Wwox Deficiency Causes Downregulation of Prosurvival ERK Signaling and Abnormal Homeostatic Responses in Mouse Skin
Source: Front Cell Dev Biol. 2020 Oct 27;8:558432. doi: 10.3389/fcell.2020.558432 (PMC7652735; doi:10.3389/fcell.2020.558432)

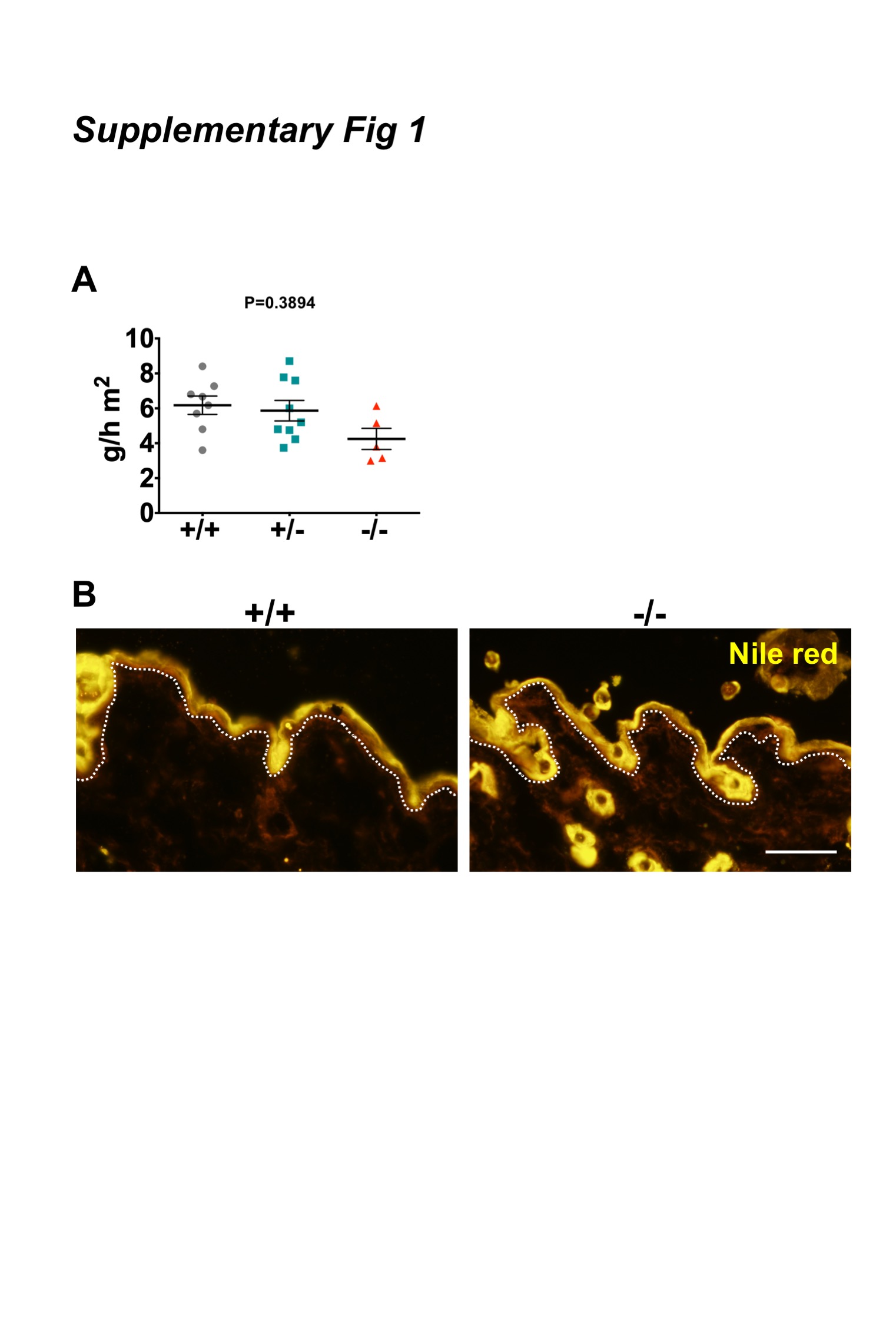

Supplement: Supplementary file 1 [file Image_1.JPEG]

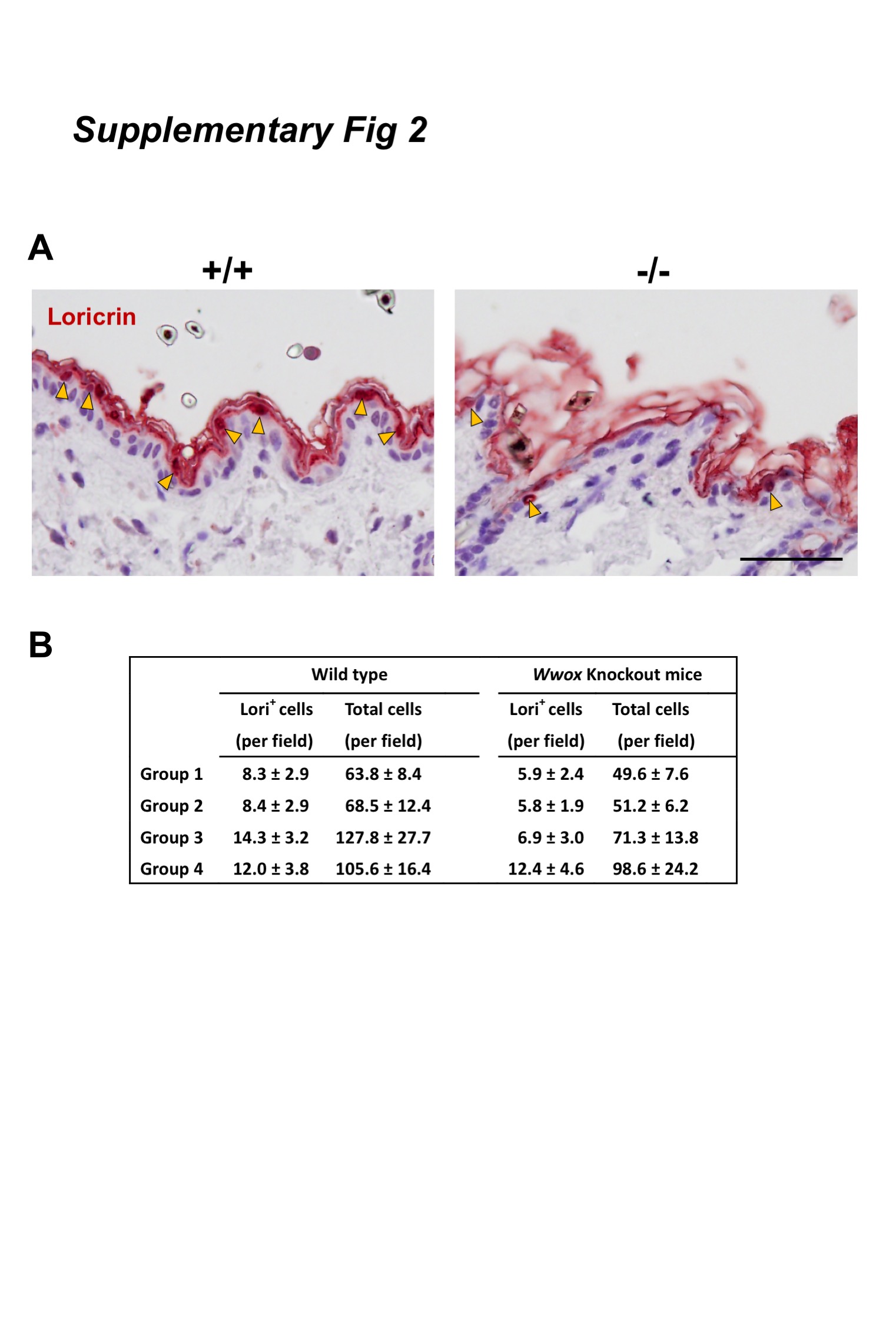

Supplement: Supplementary file 2 [file Image_2.JPEG]

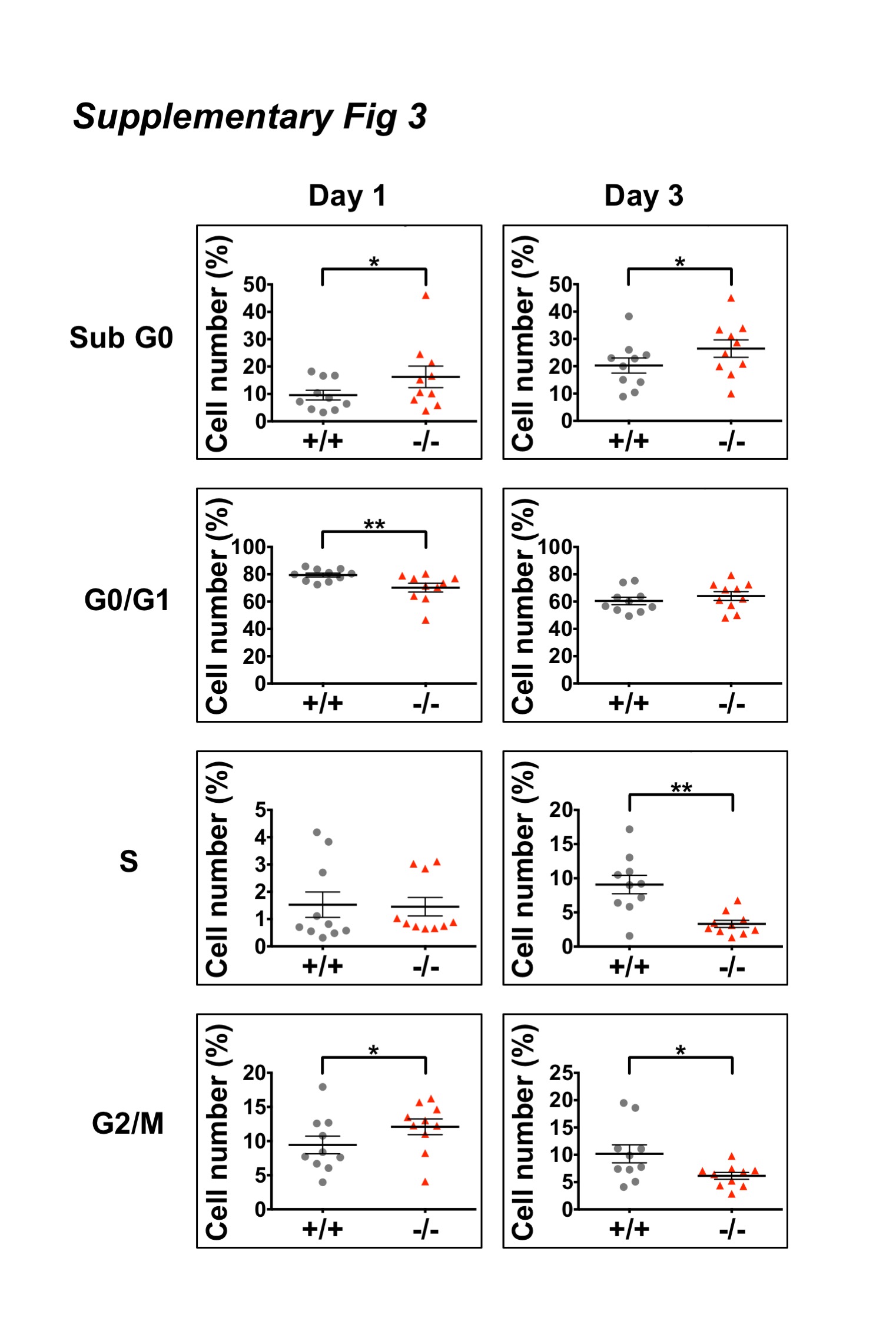

Supplement: Supplementary file 3 [file Image_3.JPEG]

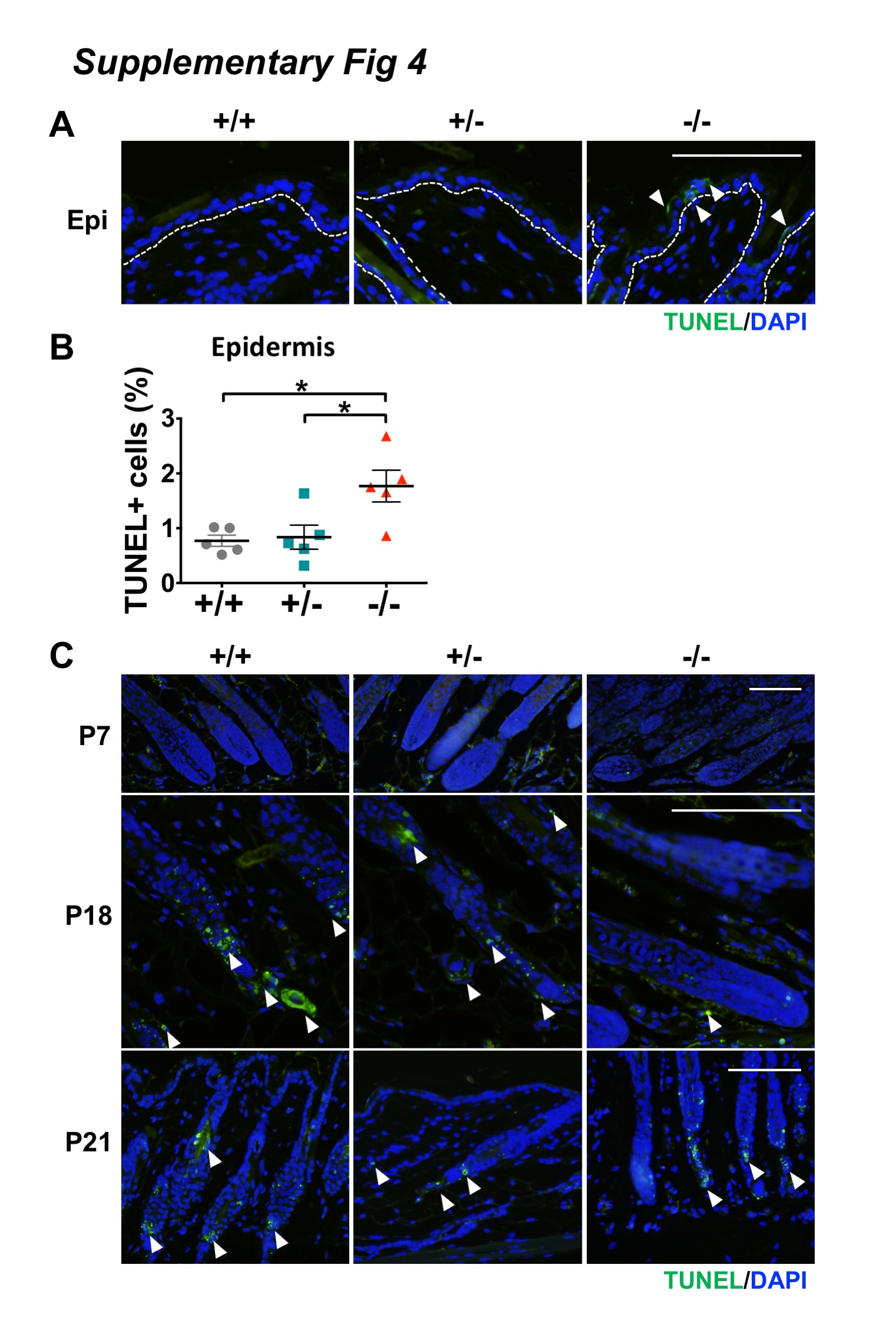

Supplement: Supplementary file 4 [file Image_4.JPEG]

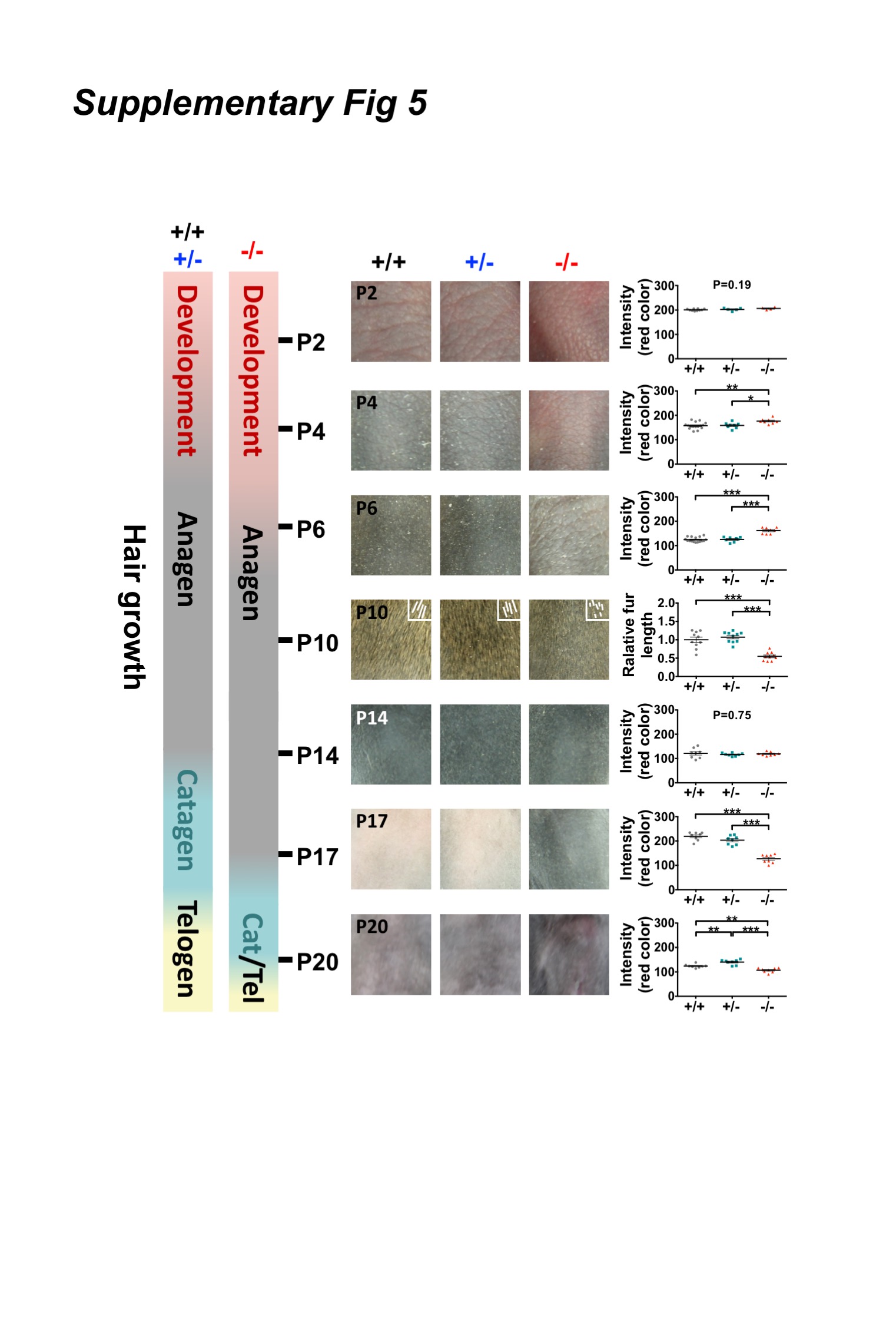

Supplement: Supplementary file 5 [file Image_5.JPEG]

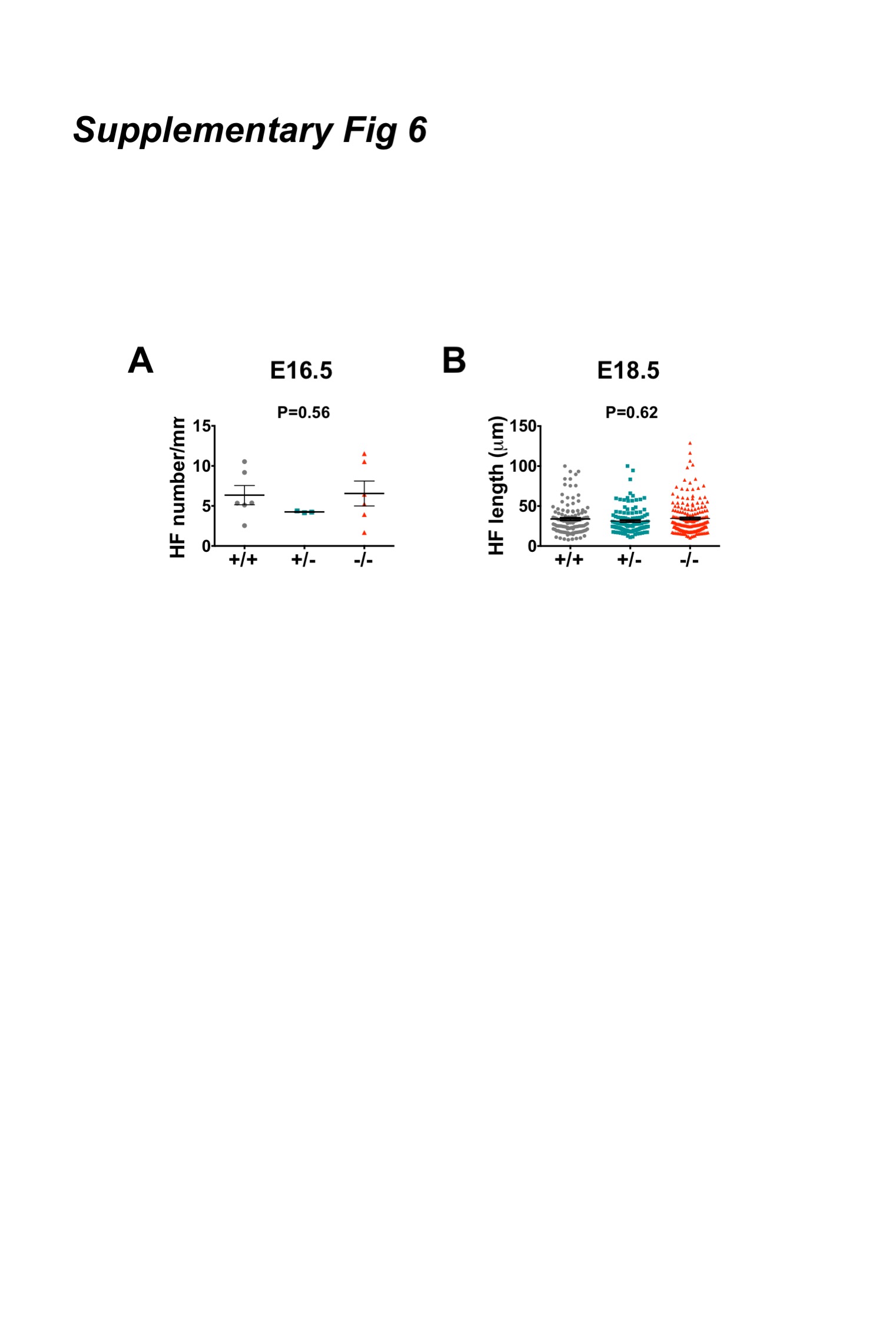

Supplement: Supplementary file 6 [file Image_6.JPEG]

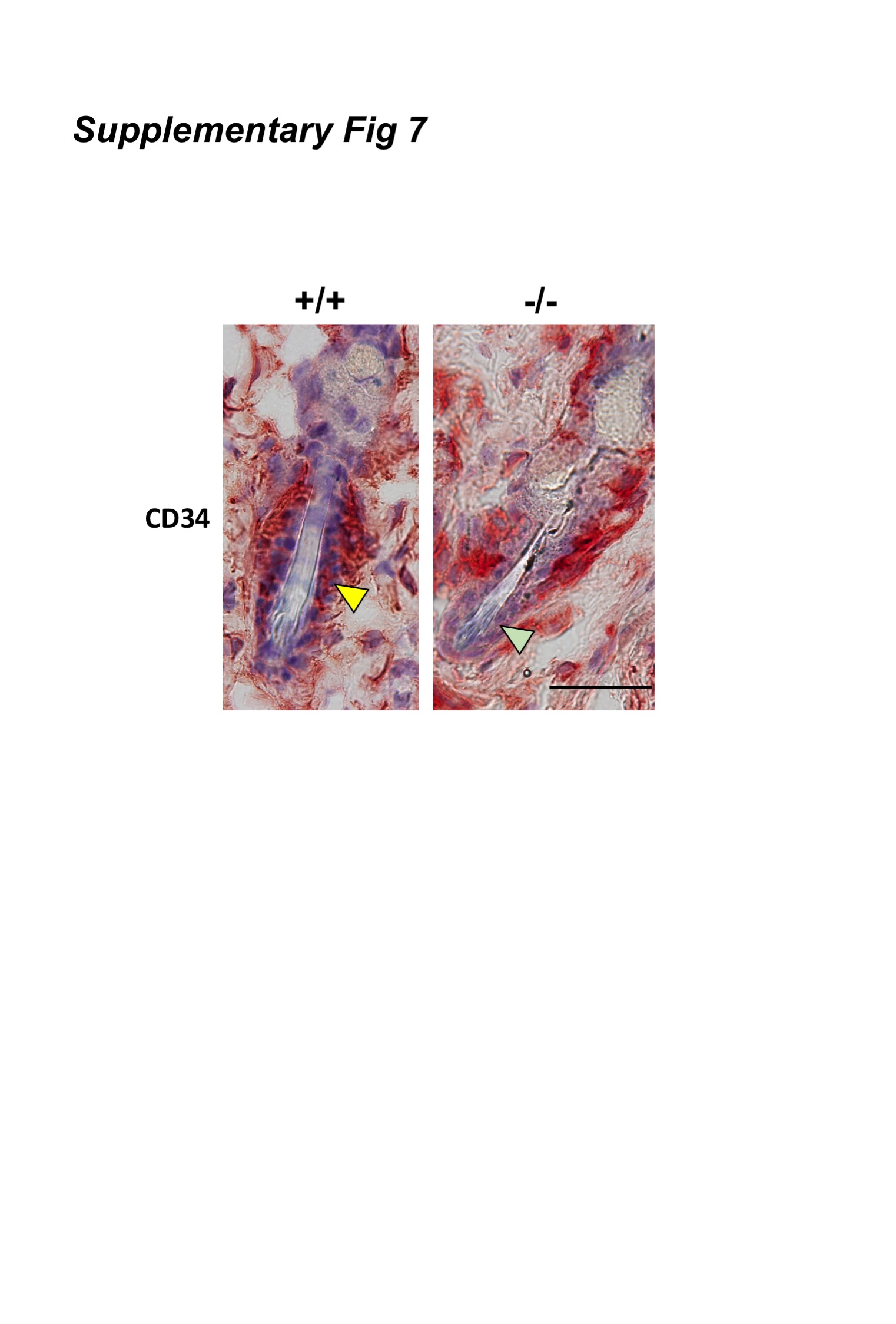

Supplement: Supplementary file 7 [file Image_7.JPEG]

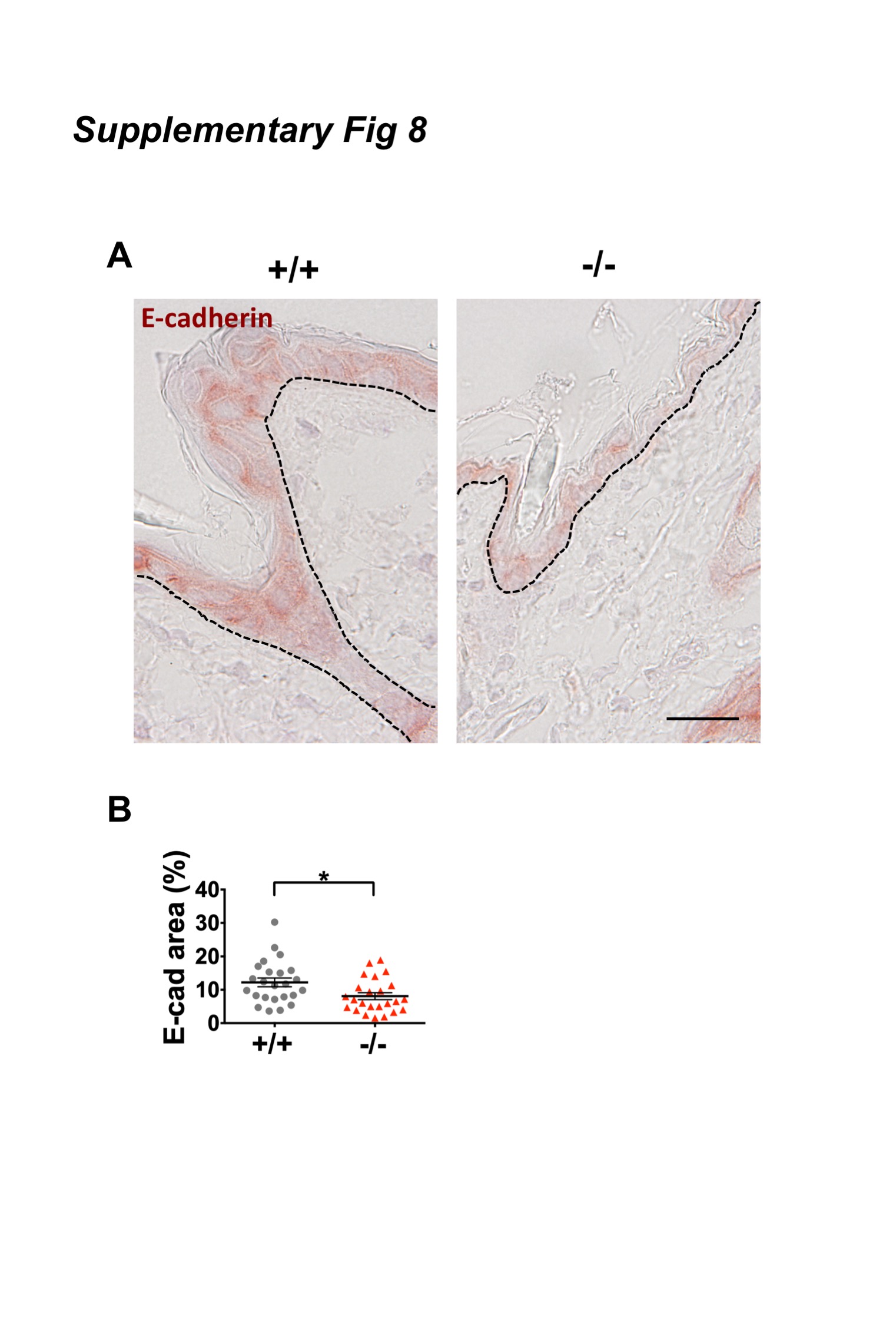

Supplement: Supplementary file 8 [file Image_8.JPEG]

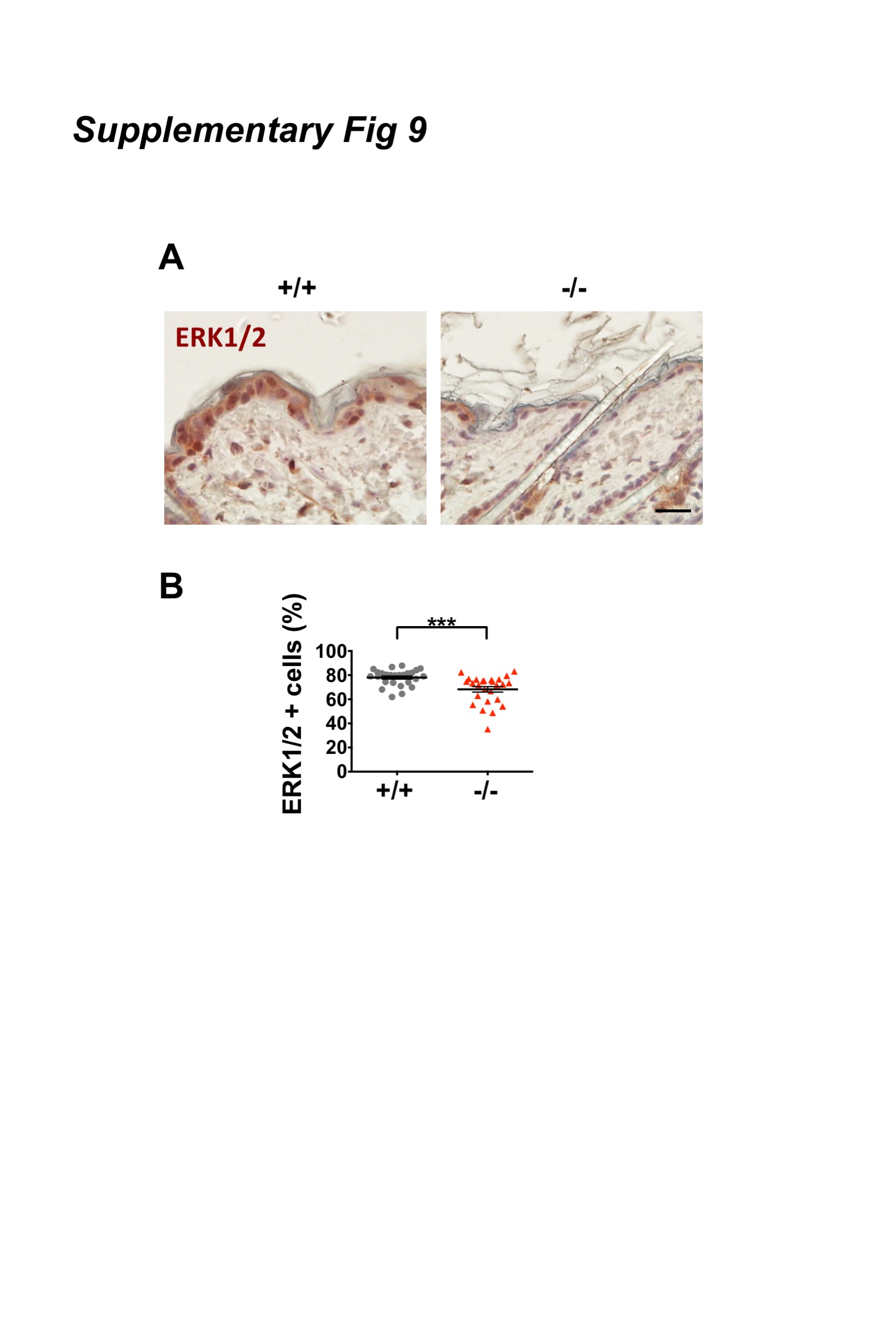

Supplement: Supplementary file 9 [file Image_9.JPEG]

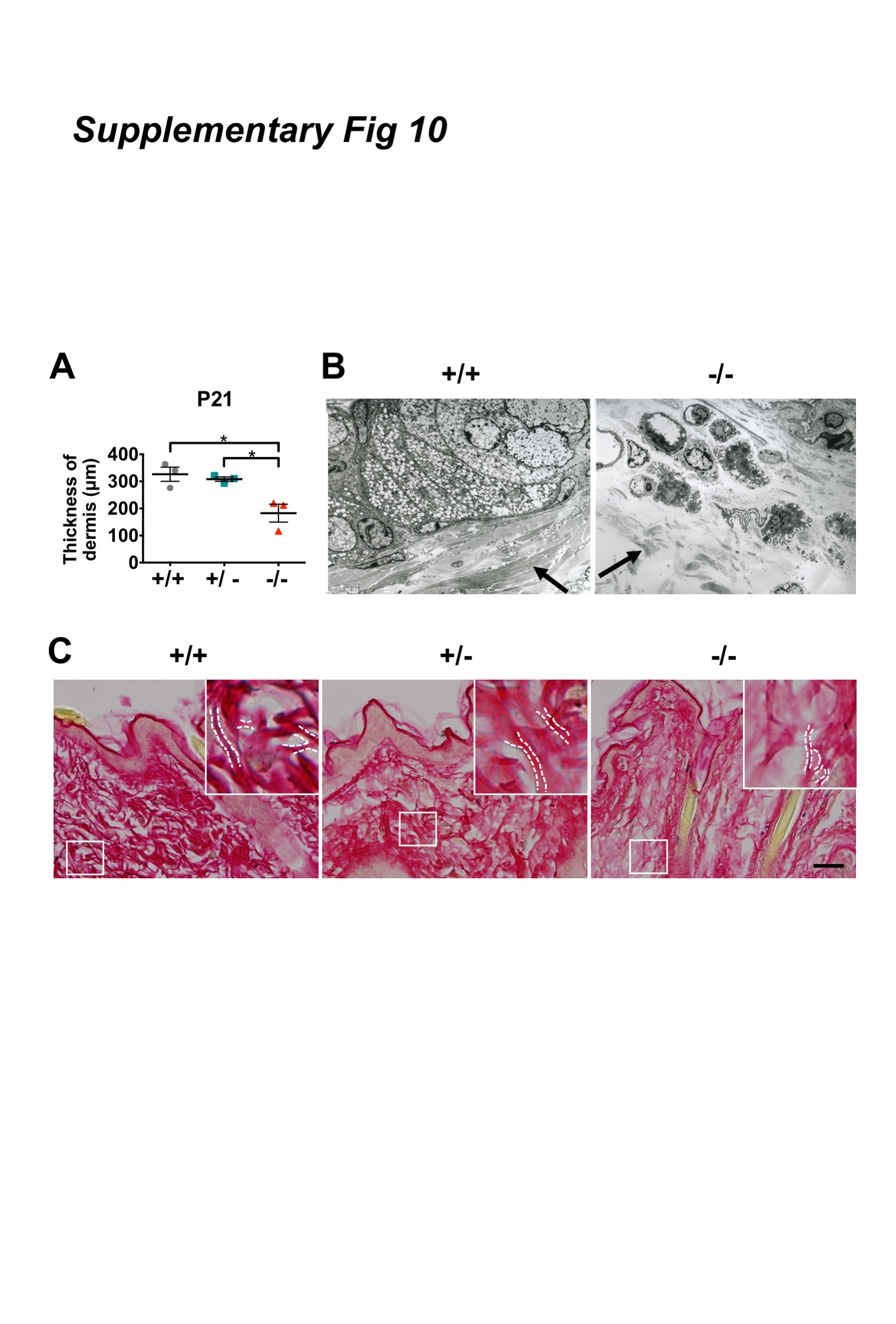

Supplement: Supplementary file 10 [file Image_10.JPEG]
