# Supplementary material for: Wwox Deficiency Causes Downregulation of Prosurvival ERK Signaling and Abnormal Homeostatic Responses in Mouse Skin
Source: Front Cell Dev Biol. 2020 Oct 27;8:558432. doi: 10.3389/fcell.2020.558432 (PMC7652735; doi:10.3389/fcell.2020.558432)
Supplement: Supplementary file 11 [file Data_Sheet_1.PDF]

SUPPLEMENTARY INFORMATION

Wwox deficiency causes downregulation of prosurvival ERK signaling and abnormal homeostatic responses in mouse skin

Ying-Tsen Chou[1], Feng-Jie Lai[2,3], Nan-Shan Chang[4,5], and Li-Jin Hsu[1,6,*]

[1]Institute of Basic Medical Sciences, College of Medicine, National Cheng Kung University, Tainan, Taiwan
[2]Department of Dermatology, Chimei Medical Center, Tainan, Taiwan
[3]Center for General Education, Southern Taiwan University of Science and Technology, Tainan, Taiwan
[4]Institute of Molecular Medicine, College of Medicine, National Cheng Kung University, Tainan, Taiwan
[5]Graduate Institute of Biomedical Sciences, College of Medicine, China Medical University, Taichung, Taiwan.
[6]Department of Medical Laboratory Science and Biotechnology, College of Medicine, National Cheng Kung University, Tainan, Taiwan

Figure S1 Water loss and lipid contents in mouse epidermis. **(A)** Transepidermal water loss (TEWL) was measured on the skin surface of *Wwox*$^{+/+}$ (n=8), *Wwox*$^{+/-}$ (n=9) and *Wwox*$^{-/-}$ (n=5) mice at P21. Data are presented as mean ± standard error of the mean (SEM). One-way ANOVA and post hoc Tukey tests were performed for statistical analysis. **(B)** Nile red staining of *Wwox*$^{+/+}$ and *Wwox*$^{-/-}$ mouse skin tissue sections at P21. Dotted white lines indicate the basement membrane. Scale bar = 20 μm.

Figure S2 Loricrin expression in wild-type and *Wwox* knockout mouse epidermis. **(A)** Immunohistochemical staining of *Wwox*$^{+/+}$ and *Wwox*$^{-/-}$ mouse skin tissue sections for loricrin. Yellow arrowheads indicate loricrin-positive keratinocytes. Scale bar = 20 μm. **(B)** Numbers of loricrin-positive cells and total cells in wild-type and *Wwox*$^{-/-}$ mouse epidermal tissues. Data are presented as mean ± SEM.

Figure S3 Cell cycle distribution of *Wwox*$^{+/+}$ and *Wwox*$^{-/-}$ mouse primary keratinocytes. *Wwox*$^{+/+}$ and *Wwox*$^{-/-}$ mouse keratinocytes were cultured *in vitro* for 1 or 3 days, and subjected to propidium iodide staining and flow cytometric analysis (n=10). Data are presented as mean ± SEM. Paired t-test was performed for statistical analysis. *$P < 0.05$, **$P < 0.01$.

Figure S4 Increased apoptotic epidermal keratinocytes and delayed hair cycle progression in *Wwox*$^{-/-}$ mice. **(A)** TUNEL assay was performed using *Wwox*$^{+/+}$, *Wwox*$^{+/-}$ and *Wwox*$^{-/-}$ mouse skin tissues at P21. Green color represents TUNEL-positive apoptotic cells in the epidermis (arrowheads). Nuclei were stained with DAPI (blue color). Dotted lines indicate the basement membrane. **(B)** Epidermal TUNEL-positive keratinocytes of *Wwox*$^{+/+}$, *Wwox*$^{+/-}$ and *Wwox*$^{-/-}$ mice at P21 were quantified (n=5). Data are presented as mean ± SEM. One-way ANOVA and post hoc Tukey tests were performed for statistical analysis. * $P < 0.05$. **(C)** TUNEL assay was performed using *Wwox*$^{+/+}$, *Wwox*$^{+/-}$ and *Wwox*$^{-/-}$ mouse epidermal tissues at P7, P18 and P21. White arrowheads indicate TUNEL-positive apoptotic cells in the hair follicle regions (green). Nuclei were stained with DAPI (blue). Scale bar = 100 μm.

Figure S5 *Wwox* depletion delays first hair cycle progression in mice. The bars in the left panel indicate the timetables of the first hair cycle progression in *Wwox*$^{+/+}$, *Wwox*$^{+/-}$ and *Wwox*$^{-/-}$ mice. The developing skin of mouse littermates was photographed continually from P2 to P20. The enlarged mouse back skin appearances from **Figure 4** are shown in the middle. The right panels indicate skin color histograms corresponding to red monochrome. From P2 to P10, the pink color disappears due to hair growth. For quantification analysis, the skin

color intensities from four areas/per mouse were analyzed at P2, and eight at P4, P6 and P14~20. At P10, the hair length was illustrated using white lines (see inserts) and quantified (10 hair fibers/per mouse). From P14 to P20, mice were shaved to show the hair cycle stages. The grey color of mouse skin depicts the growing stage of hair follicles (anagen), while the pink color indicates catagen or telogen of the hair cycle. Data are presented as mean ± SEM. One-way ANOVA and post hoc Tukey tests were performed for statistical analysis. *$P < 0.05$, **$P < 0.01$, ***$P < 0.001$.

Figure S6 Mouse hair follicle (HF) development at embryonic stages. **(A)** HF numbers were determined at embryonic day (E) 16.5 (*Wwox*$^{+/+}$, n=6; *Wwox*$^{+/-}$, n=3; *Wwox*$^{-/-}$, n=6). **(B)** HF length was quantified at E18.5. The numbers of HFs and mice analyzed (HFs/mice) were as the follows: *Wwox*$^{+/+}$ (125/6), *Wwox*$^{+/-}$ (120/3), and *Wwox*$^{-/-}$ (190/6). Data are presented as mean ± SEM. One-way ANOVA and post hoc Tukey tests were performed for statistical analysis.

Figure S7 Decreased CD34 expression in *Wwox*$^{-/-}$ mouse HF. CD34 expression was analyzed using frozen *Wwox*$^{+/+}$ and *Wwox*$^{-/-}$ mouse skin tissue sections at P21 by immunohistochemistry. The yellow arrowhead indicates CD34-positive HFSCs in the bulge area and the green arrowhead indicates weak expression of CD34. Nuclei were stained with hematoxylin. Scale bar = 20 µm.

Figure S8 Decreased E-cadherin expression in *Wwox*$^{-/-}$ mouse epidermal keratinocytes. **(A)** E-cadherin expression in *Wwox*$^{+/+}$ and *Wwox*$^{-/-}$ mouse skin tissue sections at P21 was examined by immunohistochemistry. Nuclei were stained with hematoxylin. Dotted lines indicate the basement membrane. Scale bar = 20 µm. **(B)** Quantification of E-cadherin expression in *Wwox*$^{+/+}$ and *Wwox*$^{-/-}$ mouse epidermal keratinocytes (24 regions/3 mice). All data are presented as mean ± SEM. Student's t-test was performed for statistical analysis. *$P < 0.05$.

Figure S9 Decreased ERK expression in *Wwox*$^{-/-}$ mouse epidermal keratinocytes. **(A)** Immunohistochemical staining of *Wwox*$^{+/+}$ and *Wwox*$^{-/-}$ mouse epidermal tissue sections for ERK1/2 was performed (n=3). Nuclei were stained with hematoxylin. Scale bar = 20 µm. **(C)** Quantification results of ERK1/2 expression from 26 areas are shown. All data are presented as mean ± SEM. Student's t-test was performed for statistical analysis. ***$P < 0.001$.

Figure S10 Reduction in collagen contents and fiber thickness in *Wwox*$^{-/-}$ mouse dermal tissues. **(A)** Quantification of *Wwox*$^{+/+}$, *Wwox*$^{+/-}$ and *Wwox*$^{-/-}$ mouse dermis thickness at P21 (n=3). **(B)** Collagen fibers (arrows) in *Wwox*$^{+/+}$ and *Wwox*$^{-/-}$ mouse dermal tissues are examined by transmission electron microscopy. The magnification is 30,000x. **(C)** Collagen fibers were examined using Sirius red staining. The enlarged images (upper right) are from the lower boxed areas. Collagen bundles are outlined by white dotted lines. Scale bar = 20 μm.
